# Supplementary figures and images for: Caspase-2 protects against ferroptotic cell death
Source: Cell Death Dis. 2024 Mar 1;15(3):182. doi: 10.1038/s41419-024-06560-6 (PMC10907636; doi:10.1038/s41419-024-06560-6)

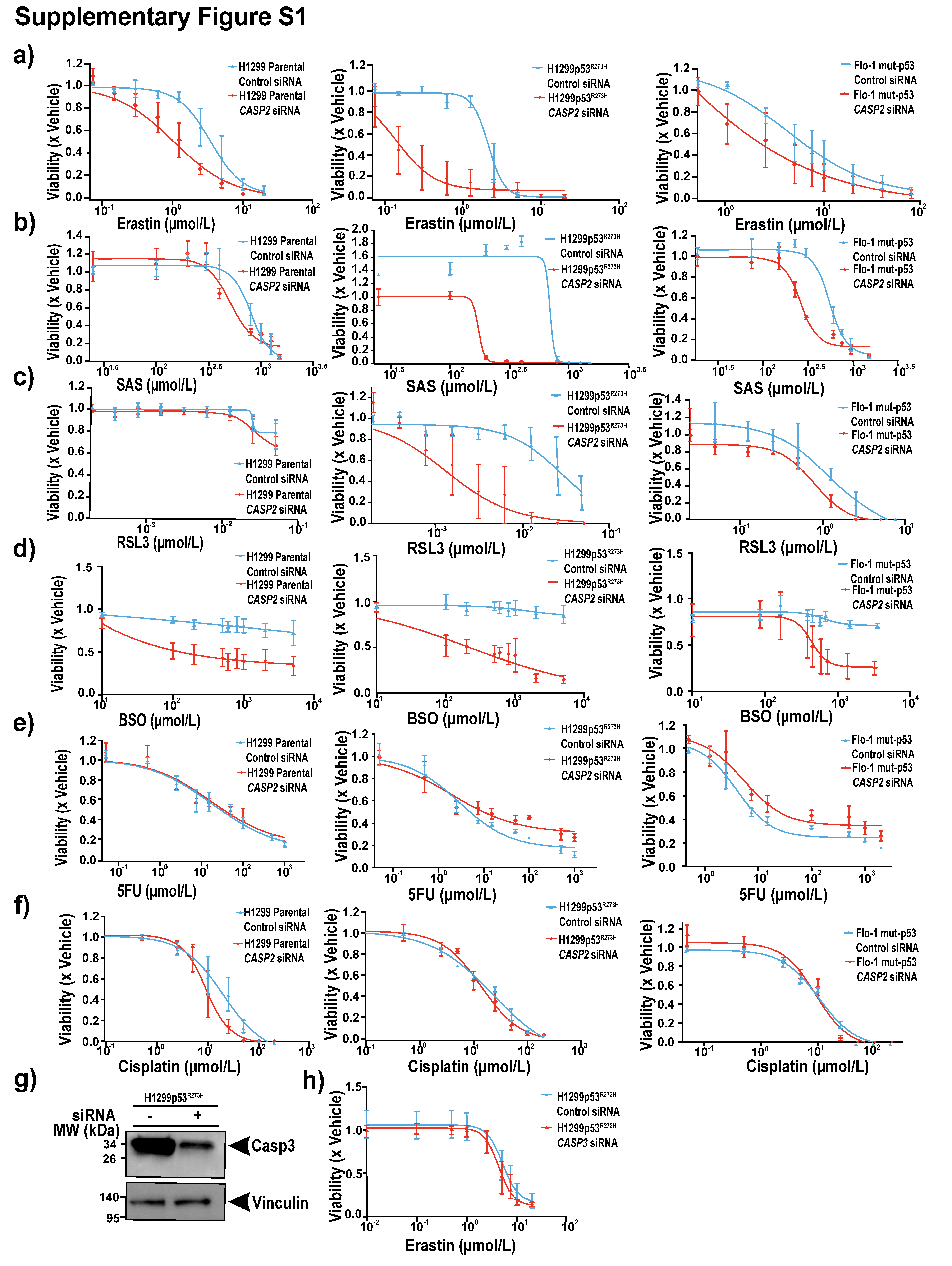

Supplement: Supplementary file 1 — Supplementary Figure S1 [file 41419_2024_6560_MOESM1_ESM.tif]

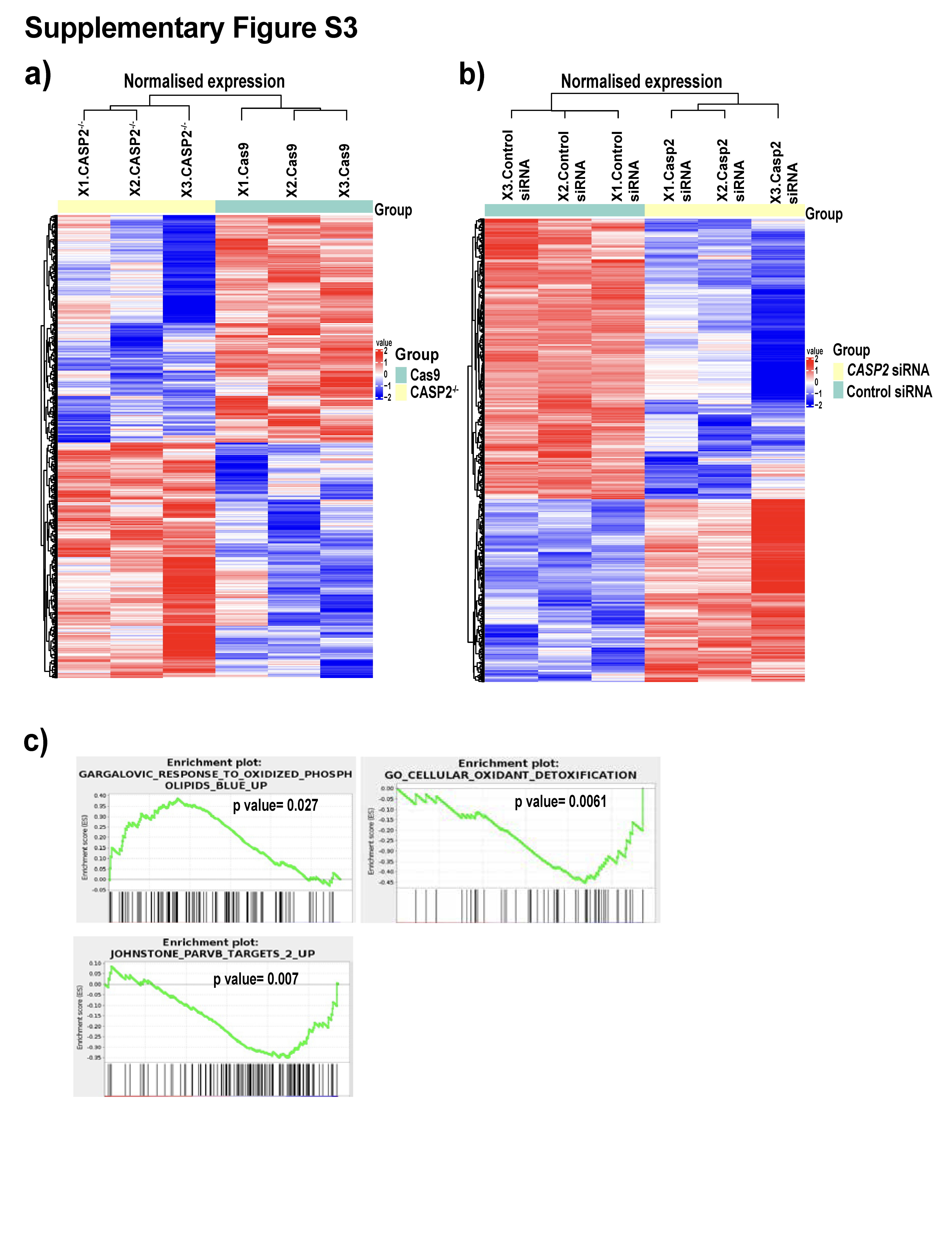

Supplement: Supplementary file 3 — Supplementary Figure S3 [file 41419_2024_6560_MOESM3_ESM.tif]

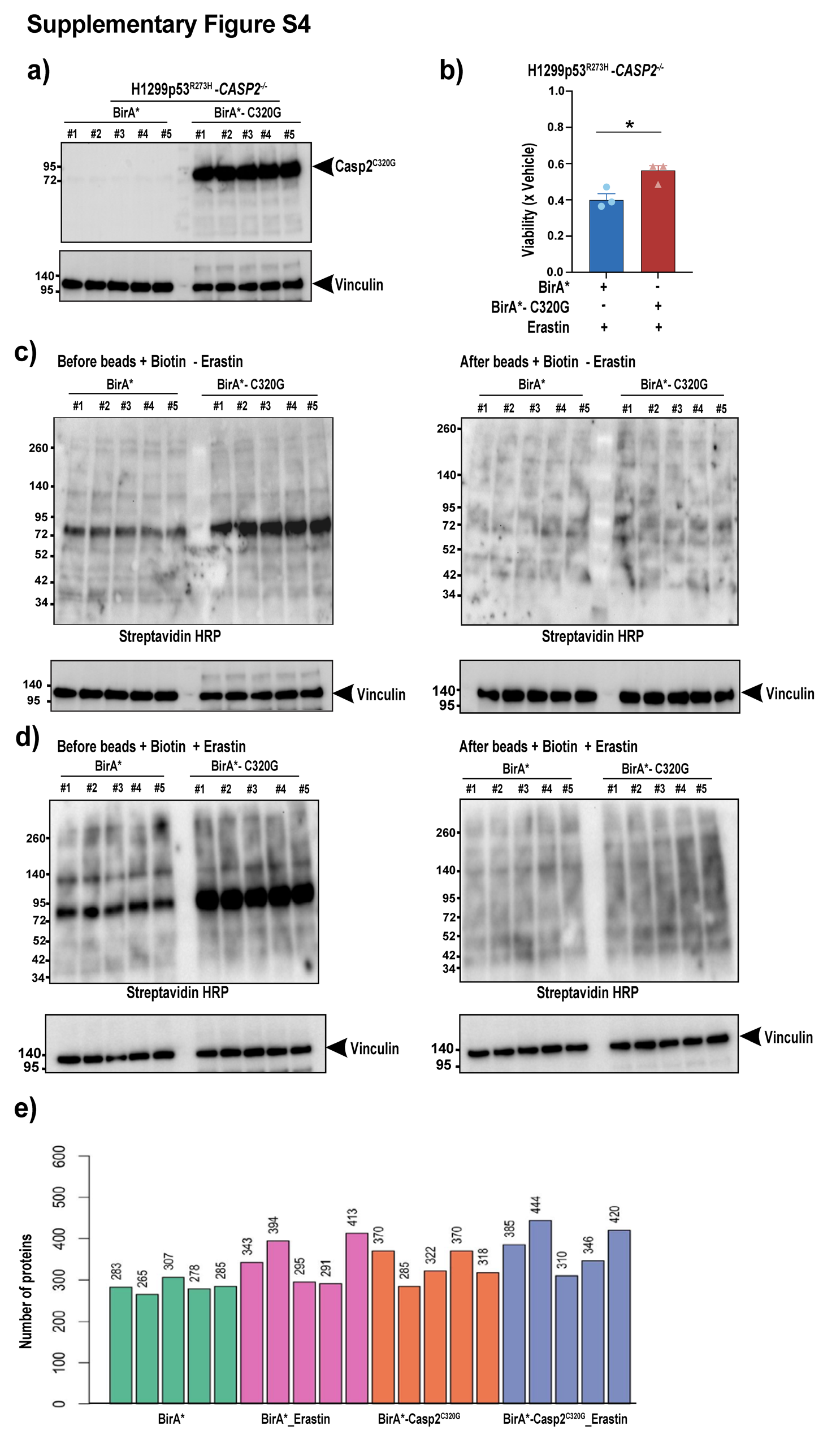

Supplement: Supplementary file 4 — Supplementary Figure S4 [file 41419_2024_6560_MOESM4_ESM.tif]

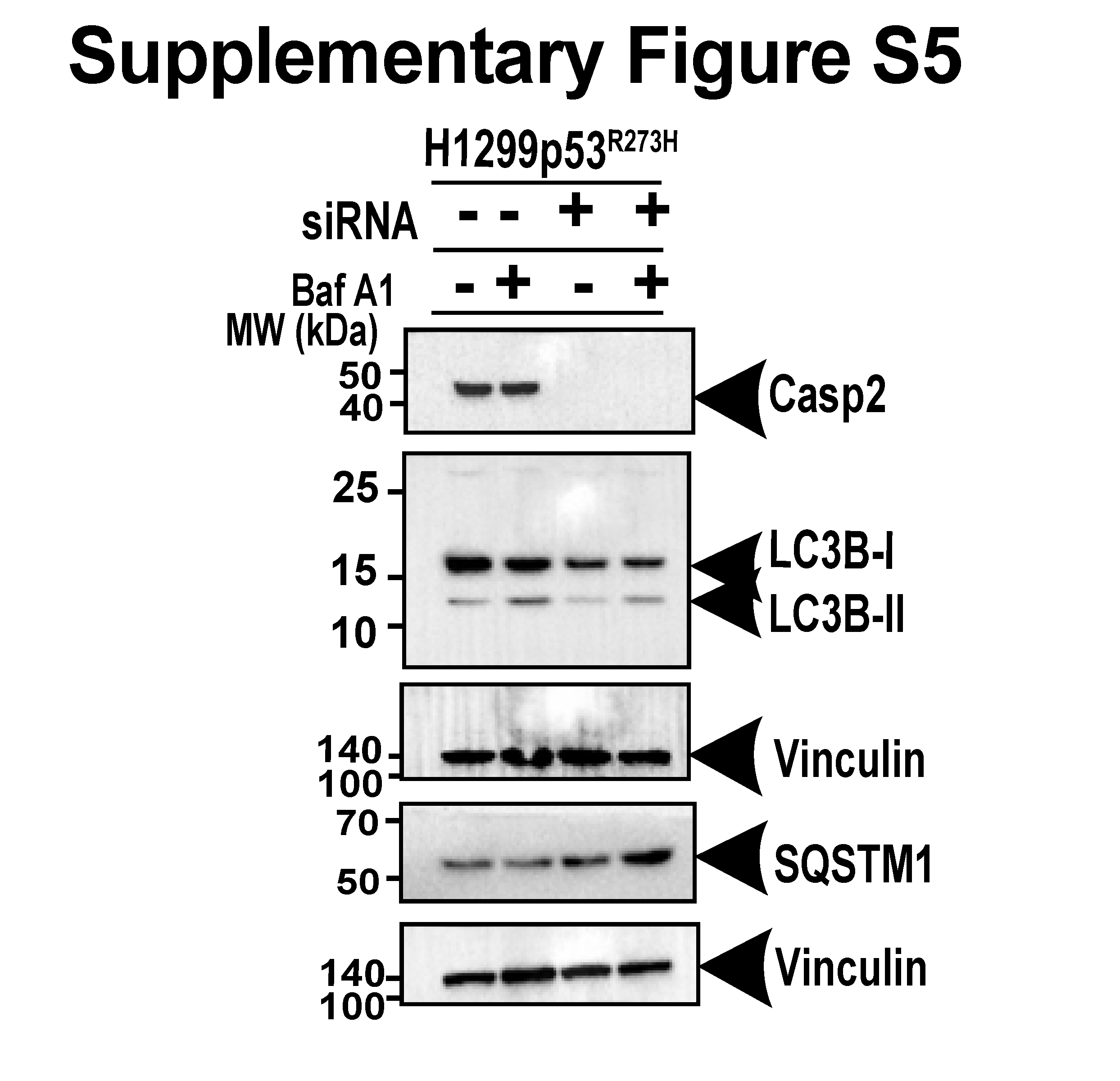

Supplement: Supplementary file 5 — Supplementary Figure S5 [file 41419_2024_6560_MOESM5_ESM.tif]
