# Supplementary material for: Caspase-2 protects against ferroptotic cell death
Source: Cell Death Dis. 2024 Mar 1;15(3):182. doi: 10.1038/s41419-024-06560-6 (PMC10907636; doi:10.1038/s41419-024-06560-6)
Supplement: Supplementary file 7 — Supplementary Table 1 [file 41419_2024_6560_MOESM7_ESM.pdf]

Supplementary table 1

List of all proteins identified by Bio-ID

UniProt	Genes	Protein Name	BirA vs BirA_C320G_logFC	BirA vs BirA_C320G_adj.P.Val	Significant BirA vs BirA_C320G	BirA_Erastin vs BirA_C320G_Erastin_logFC	BirA_Erastin vs BirA_C320G_Erastin_adj.P.Val	Significant BirA_Erastin vs BirA_C320G_Erastin
P17066	HSPA6	Heat shock 70 kDa protein 6 (Heat shock 70 kDa protein B')	-3.37	8.01E-06	Down	-1.45	4.35E-02	Down
P42575	CASP2	Caspase-2 (CASP-2) (EC 3.4.22.55) (Neural precursor cell expressed developmentally down-regulated protein 2) (NEDD-2) (Protease ICH-1) [Cleaved into: Caspase-2 subunit p18; Caspase-2 subunit p13; Caspase-2 subunit p12]	-6.07	2.09E-08	Down	-5.92	2.70E-08	Down
Q96JH7	VCPIP1	Deubiquitinating protein VCPIP1 (EC 3.4.19.12) (Valosin-containing protein p97/p47 complex-interacting protein 1) (Valosin-containing protein p97/p47 complex-interacting protein p135) (VCP/p47 complex-interacting 135-kDa protein)	-2.51	3.73E-10	Down	-2.63	2.56E-10	Down
Q9BR76	CORO1B	Coronin-1B (Coronin-2)	-1.52	1.03E-05	Down	-1.14	5.05E-04	Down
P50990	CCT8	T-complex protein 1 subunit theta (TCP-1-theta) (CCT-theta) (Chaperonin containing T-complex polypeptide 1 subunit 8) (Renal carcinoma antigen NY-REN-15)	-2.25	1.06E-11	Down	-2.13	5.51E-11	Down
Q02543	RPL18A	60S ribosomal protein L18a (Large ribosomal subunit protein eL20)	-1.56	2.00E-05	Down	-2.34	5.95E-08	Down
Q9Y265	RUVBL1	RuvB-like 1 (EC 3.6.4.12) (49 kDa TATA box-binding protein-interacting protein) (49 kDa TBP-interacting protein) (54 kDa erythrocyte cytosolic protein) (ECP-54) (INO80 complex subunit H) (Nuclear matrix protein 238) (NMP 238) (Pontin 52) (TIP49a) (TIP60-associated protein 54-alpha) (TAP54-alpha)	-3.05	6.53E-09	Down	-2.84	1.82E-08	Down
P46108	CRK	Adapter molecule crk (Proto-oncogene c-Crk) (p38)	-2.86	1.02E-07	Down	-2.64	2.88E-07	Down
Q6Y7W6	GIGYF2	GRB10-interacting GYF protein 2 (PERQ amino acid-rich with GYF domain-containing protein 2) (Trinucleotide repeat-containing gene 15 protein)	-1.41	4.82E-03	Down	-1.18	3.04E-02	Down
P11142	HSPA8	Heat shock cognate 71 kDa protein (EC 3.6.4.10) (Heat shock 70 kDa protein 8) (Lipopolysaccharide-associated protein 1) (LAP-1) (LPS-associated protein 1)	-2.50	1.06E-11	Down	-2.03	2.85E-10	Down
P25685	DNAJB1	DnaJ homolog subfamily B member 1 (DnaJ protein homolog 1) (Heat shock 40 kDa protein 1) (HSP40) (Heat shock protein 40) (Human DnaJ protein 1) (hDj-1)	-1.15	1.17E-02	Down	-1.50	2.13E-03	Down
Q9Y266	NUDC	Nuclear migration protein nudC (Nuclear distribution protein C homolog)	-1.76	1.02E-07	Down	-2.01	1.82E-08	Down
P46109	CRKL	Crk-like protein	-1.79	9.88E-07	Down	-1.55	7.64E-06	Down
Q96HC4	PDLIM5	PDZ and LIM domain protein 5 (Enigma homolog) (Enigma-like PDZ and LIM domains protein)	-1.37	1.17E-02	Down	-1.27	3.04E-02	Down
P04637	TP53	Cellular tumor antigen p53 (Antigen NY-CO-13) (Phosphoprotein p53) (Tumor suppressor p53)	-1.31	1.17E-02	Down	-1.34	1.73E-02	Down
Q7Z794	KRT77	Keratin, type II cytoskeletal 1b (Cytokeratin-1B) (CK-1B) (Keratin-77) (K77) (Type-II keratin Kb39)	0.19	8.63E-01	Not Sig.	-1.64	1.66E-02	Down
P78358	CTAG1A	Cancer/testis antigen 1 (Autoimmunogenic cancer/testis antigen NY-ESO-1) (Cancer/testis antigen 6.1) (CT6.1) (L antigen family member 2) (LAGE-2)	-0.79	2.01E-01	Not Sig.	-1.27	2.53E-02	Down
Q13501	SQSTM1	Sequestosome-1 (EBI3-associated protein of 60 kDa) (EBIAP) (p60) (Phosphotyrosine-independent ligand for the Lck SH2 domain of 62 kDa) (Ubiquitin-binding protein p62)	-1.17	9.27E-02	Not Sig.	-1.60	2.36E-02	Down

P07355	ANXA2	Annexin A2 (Annexin II) (Annexin-2) (Calpactin I heavy chain) (Calpactin-1 heavy chain) (Chromobindin-8) (Lipocortin II) (Placental anticoagulant protein IV) (PAP-IV) (Protein I) (p36)	0.16	7.15E-01	Not Sig.	-0.09	8.23E-01	Not Sig.
P00390	GSR	Glutathione reductase, mitochondrial (GR) (GRase) (EC 1.8.1.7)	-0.03	9.90E-01	Not Sig.	0.10	9.58E-01	Not Sig.
P09382	LGALS1	Galectin-1 (Gal-1) (14 kDa laminin-binding protein) (HLBP14) (14 kDa lectin) (Beta-galactoside-binding lectin L-14-I) (Galaptin) (HBL) (HPL) (Lactose-binding lectin 1) (Lectin galactoside-binding soluble 1) (Putative MAPK-activating protein PM12) (S-Lac lectin 1)	0.23	7.30E-01	Not Sig.	-0.24	6.79E-01	Not Sig.
Q15417	CNN3	Calponin-3 (Calponin, acidic isoform)	-0.03	9.72E-01	Not Sig.	0.13	8.36E-01	Not Sig.
P25398	RPS12	40S ribosomal protein S12 (Small ribosomal subunit protein eS12)	-0.03	9.87E-01	Not Sig.	-0.25	7.33E-01	Not Sig.
P09936	UCHL1	Ubiquitin carboxyl-terminal hydrolase isozyme L1 (UCH-L1) (EC 3.4.19.12) (Neuron cytoplasmic protein 9.5) (PGP 9.5) (PGP9.5) (Ubiquitin thioesterase L1)	-0.25	7.09E-01	Not Sig.	-0.20	7.41E-01	Not Sig.
P43358	MAGEA4	Melanoma-associated antigen 4 (Cancer/testis antigen 1.4) (CT1.4) (MAGE-4 antigen) (MAGE-41 antigen) (MAGE-X2 antigen)	0.28	5.70E-01	Not Sig.	0.11	8.36E-01	Not Sig.
P33316	DUT	Deoxyuridine 5'-triphosphate nucleotidohydrolase, mitochondrial (dUTPase) (EC 3.6.1.23) (dUTP pyrophosphatase)	0.60	3.85E-01	Not Sig.	0.20	8.06E-01	Not Sig.
P10599	TXN	Thioredoxin (Trx) (ATL-derived factor) (ADF) (Surface-associated sulphydryl protein) (SASP) (allergen Hom s Trx)	0.25	7.45E-01	Not Sig.	-0.20	7.84E-01	Not Sig.
P67936	TPM4	Tropomyosin alpha-4 chain (TM30p1) (Tropomyosin-4)	-0.14	8.30E-01	Not Sig.	0.51	2.30E-01	Not Sig.
P04080	CSTB	Cystatin-B (CPI-B) (Liver thiol proteinase inhibitor) (Stefin-B)	-0.56	6.91E-01	Not Sig.	0.10	9.66E-01	Not Sig.
P06748	NPM1	Nucleophosmin (NPM) (Nucleolar phosphoprotein B23) (Nucleolar protein NO38) (Numatrin)	0.20	7.30E-01	Not Sig.	0.39	4.04E-01	Not Sig.
P12268	IMPDH2	Inosine-5'-monophosphate dehydrogenase 2 (IMP dehydrogenase 2) (IMPD 2) (IMPDH 2) (EC 1.1.1.205) (Inosine-5'-monophosphate dehydrogenase type II) (IMP dehydrogenase II) (IMPDH-II)	-0.04	9.60E-01	Not Sig.	0.42	3.95E-01	Not Sig.
P61353	RPL27	60S ribosomal protein L27 (Large ribosomal subunit protein eL27)	-0.59	2.83E-01	Not Sig.	0.71	1.29E-01	Not Sig.
Q96C19	EFHD2	EF-hand domain-containing protein D2 (Swiprosin-1)	-1.21	1.40E-02	Down	-0.56	3.65E-01	Not Sig.
O15067	PFAS	Phosphoribosylformylglycinamidine synthase (FGAM synthase) (FGAMS) (EC 6.3.5.3) (Formylglycinamide ribonucleotide amidotransferase) (FGAR amidotransferase) (FGAR-AT) (Formylglycinamide ribotide amidotransferase)	-1.15	9.92E-03	Down	-0.34	5.49E-01	Not Sig.
Q9Y2Z0	SUGT1	Protein SGT1 homolog (Protein 40-6-3) (Sgt1) (Suppressor of G2 allele of SKP1 homolog)	-1.29	6.57E-04	Down	-0.68	8.89E-02	Not Sig.
Q14247	CTTN	Src substrate cortactin (Amplaxin) (Oncogene EMS1)	-1.18	2.67E-05	Down	-0.44	1.15E-01	Not Sig.
Q587J7	TDRD12	Putative ATP-dependent RNA helicase TDRD12 (EC 3.6.4.13) (ES cell-associated transcript 8 protein) (Tudor domain-containing protein 12)	-1.32	9.42E-05	Down	-0.98	3.74E-03	Not Sig.
P62979	RPS27A	Ubiquitin-40S ribosomal protein S27a (Ubiquitin carboxyl extension protein 80) [Cleaved into: Ubiquitin; 40S ribosomal protein S27a (Small ribosomal subunit protein eS31)]	-1.43	3.93E-03	Down	-0.97	7.20E-02	Not Sig.
O95817	BAG3	BAG family molecular chaperone regulator 3 (BAG-3) (Bcl-2-associated athanogene 3) (Bcl-2-binding protein Bis) (Docking protein CAIR-1)	-1.14	6.49E-04	Down	-0.55	1.14E-01	Not Sig.

P51610	HCFC1	Host cell factor 1 (HCF) (HCF-1) (C1 factor) (CFF) (VCAF) (VP16 accessory protein) [Cleaved into: HCF N-terminal chain 1; HCF N-terminal chain 2; HCF N-terminal chain 3; HCF N-terminal chain 4; HCF N-terminal chain 5; HCF N-terminal chain 6; HCF C-terminal chain 1; HCF C-terminal chain 2; HCF C-terminal chain 3; HCF C-terminal chain 4; HCF C-terminal chain 5; HCF C-terminal chain 6]	-1.27	2.67E-05	Down	-0.59	5.10E-02	Not Sig.
O14641	DVL2	Segment polarity protein dishevelled homolog DVL-2 (Dishevelled-2) (DSH homolog 2)	-1.30	1.45E-04	Down	-0.72	4.30E-02	Not Sig.
Q9UHR5	SAP30BP	SAP30-binding protein (Transcriptional regulator protein HCNGP)	-1.97	1.06E-03	Down	-1.23	5.61E-02	Not Sig.
O75190	DNAJB6	DnaJ homolog subfamily B member 6 (HHDJ1) (Heat shock protein J2) (HSJ-2) (MRJ) (MSJ-1)	-1.13	2.60E-02	Down	-0.70	2.04E-01	Not Sig.
P37108	SRP14	Signal recognition particle 14 kDa protein (SRP14) (18 kDa Alu RNA-binding protein)	0.57	1.38E-01	Not Sig.	0.17	7.46E-01	Not Sig.
P31948	STIP1	Stress-induced-phosphoprotein 1 (STI1) (Hsc70/Hsp90-organizing protein) (Hop) (Renal carcinoma antigen NY-REN-11) (Transformation-sensitive protein IEF SSP 3521)	-0.96	2.83E-04	Not Sig.	-0.42	1.19E-01	Not Sig.
P49327	FASN	Fatty acid synthase (EC 2.3.1.85) (Type I fatty acid synthase) [Includes: [Acyl-carrier-protein] S-acetyltransferase (EC 2.3.1.38); [Acyl-carrier-protein] S-malonyltransferase (EC 2.3.1.39); 3-oxoacyl-[acyl-carrier-protein] synthase (EC 2.3.1.41); 3-oxoacyl-[acyl-carrier-protein] reductase (EC 1.1.1.100); 3-hydroxyacyl-[acyl-carrier-protein] dehydratase (EC 4.2.1.59); Enoyl-[acyl-carrier-protein] reductase (EC 1.3.1.39); Acyl-[acyl-carrier-protein] hydrolase (EC 3.1.2.14)]	-0.80	4.69E-04	Not Sig.	-0.39	1.01E-01	Not Sig.
P62258	YWHAE	14-3-3 protein epsilon (14-3-3E)	0.29	7.63E-01	Not Sig.	0.31	7.24E-01	Not Sig.
P02768	ALB	Albumin	0.02	9.90E-01	Not Sig.	0.40	6.77E-01	Not Sig.
P35232	PHB	Prohibitin	0.10	9.30E-01	Not Sig.	0.29	6.99E-01	Not Sig.
Q15149	PLEC	Plectin (PCN) (PLTN) (Hemidesmosomal protein 1) (HD1) (Plectin-1)	0.12	7.05E-01	Not Sig.	0.31	1.49E-01	Not Sig.
P62851	RPS25	40S ribosomal protein S25 (Small ribosomal subunit protein eS25)	-0.13	8.63E-01	Not Sig.	0.74	1.15E-01	Not Sig.
P62910	RPL32	60S ribosomal protein L32 (Large ribosomal subunit protein eL32)	-0.06	9.51E-01	Not Sig.	-0.12	8.50E-01	Not Sig.
P78527	PRKDC	DNA-dependent protein kinase catalytic subunit (DNA-PK catalytic subunit) (DNA-PKcs) (EC 2.7.11.1) (DNPK1) (p460)	-0.05	9.52E-01	Not Sig.	0.10	8.57E-01	Not Sig.
Q9BUF5	TUBB6	Tubulin beta-6 chain (Tubulin beta class V)	0.47	1.31E-01	Not Sig.	-0.04	9.55E-01	Not Sig.
Q08211	DHX9	ATP-dependent RNA helicase A (EC 3.6.4.13) (DEAH box protein 9) (DExH-box helicase 9) (Leukophysin) (LKP) (Nuclear DNA helicase II) (NDH II) (RNA helicase A)	0.12	7.09E-01	Not Sig.	0.14	6.62E-01	Not Sig.
Q15233	NONO	Non-POU domain-containing octamer-binding protein (NonO protein) (54 kDa nuclear RNA- and DNA-binding protein) (55 kDa nuclear protein) (DNA-binding p52/p100 complex, 52 kDa subunit) (NMT55) (p54(nrb)) (p54nrb)	-0.89	1.02E-03	Not Sig.	-0.64	2.93E-02	Not Sig.
P13804	ETFA	Electron transfer flavoprotein subunit alpha, mitochondrial (Alpha-ETF)	-0.72	4.20E-02	Not Sig.	-0.75	4.35E-02	Not Sig.
P02545	LMNA	Prelamin-A/C [Cleaved into: Lamin-A/C (70 kDa lamin) (Renal carcinoma antigen NY-REN-32)]	-0.09	9.30E-01	Not Sig.	0.55	4.04E-01	Not Sig.
P05141	SLC25A5	ADP/ATP translocase 2 (ADP,ATP carrier protein 2) (ADP,ATP carrier protein, fibroblast isoform) (Adenine nucleotide translocator 2) (ANT 2) (Solute carrier family 25 member 5) [Cleaved into:	0.05	9.25E-01	Not Sig.	-0.06	8.86E-01	Not Sig.

		ADP/ATP translocase 2, N-terminally processed]						
P00558	PGK1	Phosphoglycerate kinase 1 (EC 2.7.2.3) (Cell migration-inducing gene 10 protein) (Primer recognition protein 2) (PRP 2)	-0.12	8.41E-01	Not Sig.	0.56	1.42E-01	Not Sig.
P61247	RPS3A	40S ribosomal protein S3a (Small ribosomal subunit protein eS1) (v-fos transformation effector protein) (Fte-1)	0.10	8.11E-01	Not Sig.	0.05	9.08E-01	Not Sig.
P11498	PC	Pyruvate carboxylase, mitochondrial (EC 6.4.1.1) (Pyruvic carboxylase) (PCB)	0.06	8.41E-01	Not Sig.	0.29	1.49E-01	Not Sig.
P09622	DLD	Dihydrolipoyl dehydrogenase, mitochondrial (EC 1.8.1.4) (Dihydrolipoamide dehydrogenase) (Glycine cleavage system L protein)	0.25	7.19E-01	Not Sig.	-0.38	5.12E-01	Not Sig.
P08238	HSP90AB 1	Heat shock protein HSP 90-beta (HSP 90) (Heat shock 84 kDa) (HSP 84) (HSP84)	-0.71	3.07E-03	Not Sig.	-0.33	2.21E-01	Not Sig.
Q09666	AHNAK	Neuroblast differentiation-associated protein AHNAK (Desmoyokin)	-0.16	5.31E-01	Not Sig.	0.29	1.49E-01	Not Sig.
P52272	HNRNPM	Heterogeneous nuclear ribonucleoprotein M (hnRNP M)	0.26	4.15E-01	Not Sig.	0.21	4.94E-01	Not Sig.
Q9H074	PAIP1	Polyadenylate-binding protein-interacting protein 1 (PABP-interacting protein 1) (PAIP-1) (Poly(A)-binding protein-interacting protein 1)	0.04	9.86E-01	Not Sig.	-0.29	7.58E-01	Not Sig.
P68036	UBE2L3	Ubiquitin-conjugating enzyme E2 L3 (EC 2.3.2.23) (E2 ubiquitin-conjugating enzyme L3) (L-UBC) (UbcH7) (Ubiquitin carrier protein L3) (Ubiquitin-conjugating enzyme E2-F1) (Ubiquitin-protein ligase L3)	0.07	9.30E-01	Not Sig.	0.51	2.21E-01	Not Sig.
Q04695	KRT17	Keratin, type I cytoskeletal 17 (39.1) (Cytokeratin-17) (CK-17) (Keratin-17) (K17)	0.49	7.05E-01	Not Sig.	-0.05	9.84E-01	Not Sig.
P13645	KRT10	Keratin, type I cytoskeletal 10 (Cytokeratin-10) (CK-10) (Keratin-10) (K10)	0.49	6.04E-01	Not Sig.	-0.79	3.06E-01	Not Sig.
Q96P63	SERPINB 12	Serpin B12	0.61	6.31E-01	Not Sig.	-1.31	1.32E-01	Not Sig.
P62263	RPS14	40S ribosomal protein S14 (Small ribosomal subunit protein uS11)	0.22	5.61E-01	Not Sig.	0.21	5.37E-01	Not Sig.
P04264	KRT1	Keratin, type II cytoskeletal 1 (67 kDa cytokeratin) (Cytokeratin-1) (CK-1) (Hair alpha protein) (Keratin-1) (K1) (Type-II keratin Kb1)	0.43	5.57E-01	Not Sig.	-0.62	3.06E-01	Not Sig.
P49411	TUFM	Elongation factor Tu, mitochondrial (EF-Tu) (P43)	0.48	8.16E-02	Not Sig.	0.12	7.68E-01	Not Sig.
P15880	RPS2	40S ribosomal protein S2 (40S ribosomal protein S4) (Protein LLRep3) (Small ribosomal subunit protein uS5)	0.28	5.81E-01	Not Sig.	-0.05	9.50E-01	Not Sig.
P82094	TMF1	TATA element modulatory factor (TMF) (Androgen receptor coactivator 160 kDa protein) (Androgen receptor-associated protein of 160 kDa)	-0.76	5.81E-01	Not Sig.	-1.04	3.92E-01	Not Sig.
Q14315	FLNC	Filamin-C (FLN-C) (FLNc) (ABP-280-like protein) (ABP-L) (Actin-binding-like protein) (Filamin-2) (Gamma-filamin)	0.14	7.05E-01	Not Sig.	0.05	8.91E-01	Not Sig.
P14618	PKM	Pyruvate kinase PKM (EC 2.7.1.40) (Cytosolic thyroid hormone-binding protein) (CTHBP) (Opa-interacting protein 3) (OIP-3) (Pyruvate kinase 2/3) (Pyruvate kinase muscle isozyme) (Thyroid hormone-binding protein 1) (THBP1) (Tumor M2-PK) (p58)	0.15	5.70E-01	Not Sig.	0.15	5.35E-01	Not Sig.
P19013	KRT4	Keratin, type II cytoskeletal 4 (Cytokeratin-4) (CK-4) (Keratin-4) (K4) (Type-II keratin Kb4)	-0.57	7.63E-01	Not Sig.	0.03	9.86E-01	Not Sig.
P21333	FLNA	Filamin-A (FLN-A) (Actin-binding protein 280) (ABP-280) (Alpha-filamin) (Endothelial actin-binding protein) (Filamin-1) (Non-muscle filamin)	-0.35	7.55E-02	Not Sig.	0.02	9.71E-01	Not Sig.
P15924	DSP	Desmoplakin (DP) (250/210 kDa paraneoplastic pemphigus antigen)	0.43	5.56E-01	Not Sig.	-0.08	9.39E-01	Not Sig.
P23396	RPS3	40S ribosomal protein S3 (EC 4.2.99.18) (Small ribosomal subunit protein uS3)	0.25	3.68E-01	Not Sig.	0.07	8.36E-01	Not Sig.
P59998	ARPC4	Actin-related protein 2/3 complex subunit 4 (Arp2/3 complex 20 kDa subunit) (p20-ARC)	0.24	7.64E-01	Not Sig.	0.31	6.77E-01	Not Sig.

P05165	PCCA	Propionyl-CoA carboxylase alpha chain, mitochondrial (PCCase subunit alpha) (EC 6.4.1.3) (Propanoyl-CoA:carbon dioxide ligase subunit alpha)	0.04	9.27E-01	Not Sig.	0.24	2.92E-01	Not Sig.
P40429	RPL13A	60S ribosomal protein L13a (23 kDa highly basic protein) (Large ribosomal subunit protein uL13)	0.06	9.36E-01	Not Sig.	-0.16	7.81E-01	Not Sig.
O75369	FLNB	Filamin-B (FLN-B) (ABP-278) (ABP-280 homolog) (Actin-binding-like protein) (Beta-filamin) (Filamin homolog 1) (Fh1) (Filamin-3) (Thyroid autoantigen) (Truncated actin-binding protein) (Truncated ABP)	0.03	9.60E-01	Not Sig.	0.53	5.10E-02	Not Sig.
Q8IVF2	AHNAK2	Protein AHNAK2	-0.25	3.93E-01	Not Sig.	0.13	6.77E-01	Not Sig.
P17987	TCP1	T-complex protein 1 subunit alpha (TCP-1-alpha) (CCT-alpha)	0.21	5.57E-01	Not Sig.	0.05	9.06E-01	Not Sig.
P49207	RPL34	60S ribosomal protein L34 (Large ribosomal subunit protein eL34)	0.24	7.34E-01	Not Sig.	-0.35	5.47E-01	Not Sig.
P34897	SHMT2	Serine hydroxymethyltransferase, mitochondrial (SHMT) (EC 2.1.2.1) (Glycine hydroxymethyltransferase) (Serine methylase)	0.60	2.25E-01	Not Sig.	-0.01	9.84E-01	Not Sig.
Q9HCC0	MCCC2	Methylcrotonoyl-CoA carboxylase beta chain, mitochondrial (MCCase subunit beta) (EC 6.4.1.4) (3-methylcrotonyl-CoA carboxylase 2) (3-methylcrotonyl-CoA carboxylase non-biotin-containing subunit) (3-methylcrotonyl-CoA:carbon dioxide ligase subunit beta)	-0.05	9.52E-01	Not Sig.	0.21	6.58E-01	Not Sig.
P04406	GAPDH	Glyceraldehyde-3-phosphate dehydrogenase (GAPDH) (EC 1.2.1.12) (Peptidyl-cysteine S-nitrosylase GAPDH) (EC 2.6.99.-)	0.18	6.35E-01	Not Sig.	-0.05	9.27E-01	Not Sig.
P14923	JUP	Junction plakoglobin (Catenin gamma) (Desmoplakin III) (Desmoplakin-3)	0.09	9.52E-01	Not Sig.	0.15	9.06E-01	Not Sig.
P62269	RPS18	40S ribosomal protein S18 (Ke-3) (Ke3) (Small ribosomal subunit protein uS13)	0.22	7.16E-01	Not Sig.	0.53	2.20E-01	Not Sig.
P25311	AZGP1	Zinc-alpha-2-glycoprotein (Zn-alpha-2-GP) (Zn-alpha-2-glycoprotein)	-0.07	9.72E-01	Not Sig.	-0.84	4.13E-01	Not Sig.
Q92598	HSPH1	Heat shock protein 105 kDa (Antigen NY-CO-25) (Heat shock 110 kDa protein)	-0.42	6.96E-01	Not Sig.	-0.31	7.56E-01	Not Sig.
P18583	SON	Protein SON (Bax antagonist selected in saccharomyces 1) (BASS1) (Negative regulatory element-binding protein) (NRE-binding protein) (Protein DBP-5) (SON3)	-0.17	8.63E-01	Not Sig.	-0.35	6.76E-01	Not Sig.
P55786	NPEPPS	Puromycin-sensitive aminopeptidase (PSA) (EC 3.4.11.14) (Cytosol alanyl aminopeptidase) (AAP-S)	0.54	2.51E-01	Not Sig.	-0.07	9.31E-01	Not Sig.
P23526	AHCY	Adenosylhomocysteinase (AdoHcyase) (EC 3.3.1.1) (S-adenosyl-L-homocysteine hydrolase)	0.18	8.74E-01	Not Sig.	0.85	2.92E-01	Not Sig.
P39023	RPL3	60S ribosomal protein L3 (HIV-1 TAR RNA-binding protein B) (TARBP-B) (Large ribosomal subunit protein uL3)	0.03	9.78E-01	Not Sig.	0.34	5.35E-01	Not Sig.
P18124	RPL7	60S ribosomal protein L7 (Large ribosomal subunit protein uL30)	-0.09	8.63E-01	Not Sig.	0.78	1.66E-02	Not Sig.
P35268	RPL22	60S ribosomal protein L22 (EBER-associated protein) (EAP) (Epstein-Barr virus small RNA-associated protein) (Heparin-binding protein HBp15) (Large ribosomal subunit protein eL22)	0.66	2.53E-01	Not Sig.	0.33	6.04E-01	Not Sig.
P62424	RPL7A	60S ribosomal protein L7a (Large ribosomal subunit protein eL8) (PLA-X polypeptide) (Surfeit locus protein 3)	-0.07	9.52E-01	Not Sig.	0.25	7.15E-01	Not Sig.
P62266	RPS23	40S ribosomal protein S23 (Small ribosomal subunit protein uS12)	-0.25	6.01E-01	Not Sig.	-0.37	3.65E-01	Not Sig.
P22102	GART	Trifunctional purine biosynthetic protein adenosine-3 [Includes: Phosphoribosylamine--glycine ligase (EC 6.3.4.13) (Glycinamide ribonucleotide synthetase) (GARS) (Phosphoribosylglycinamid	-0.09	8.83E-01	Not Sig.	0.09	8.53E-01	Not Sig.

		e synthetase); Phosphoribosylformylglycinamidine cyclo-ligase (EC 6.3.3.1) (AIR synthase) (AIRS) (Phosphoribosyl-aminoimidazole synthetase); Phosphoribosylglycinamide formyltransferase (EC 2.1.2.2) (5'-phosphoribosylglycinamide transformylase) (GAR transformylase) (GART)]						
P31327	CPS1	Carbamoyl-phosphate synthase [ammonia], mitochondrial (EC 6.3.4.16) (Carbamoyl-phosphate synthetase I) (CPSase I)	0.22	8.63E-01	Not Sig.	0.62	5.01E-01	Not Sig.
P22735	TGM1	Protein-glutamine gamma-glutamyltransferase K (EC 2.3.2.13) (Epidermal TGase) (Transglutaminase K) (TG(K)) (TGK) (TGase K) (Transglutaminase-1) (TGase-1)	0.24	8.63E-01	Not Sig.	-0.89	3.54E-01	Not Sig.
P62081	RPS7	40S ribosomal protein S7 (Small ribosomal subunit protein eS7)	0.06	9.52E-01	Not Sig.	0.14	8.46E-01	Not Sig.
Q9NQH7	XPNPEP3	Xaa-Pro aminopeptidase 3 (X-Pro aminopeptidase 3) (EC 3.4.11.9) (Aminopeptidase P3) (APP3)	-0.61	3.85E-01	Not Sig.	0.00	9.99E-01	Not Sig.
Q02878	RPL6	60S ribosomal protein L6 (Large ribosomal subunit protein eL6) (Neoplasm-related protein C140) (Tax-responsive enhancer element-binding protein 107) (TaxREB107)	-0.06	9.52E-01	Not Sig.	0.60	1.56E-01	Not Sig.
Q99614	TTC1	Tetratricopeptide repeat protein 1 (TPR repeat protein 1)	-0.54	4.15E-01	Not Sig.	-0.67	2.31E-01	Not Sig.
P83731	RPL24	60S ribosomal protein L24 (60S ribosomal protein L30) (Large ribosomal subunit protein eL24)	-0.01	9.94E-01	Not Sig.	0.30	6.79E-01	Not Sig.
P14866	HNRNPL	Heterogeneous nuclear ribonucleoprotein L (hnRNP L)	0.32	2.46E-01	Not Sig.	0.49	4.89E-02	Not Sig.
Q15366	PCBP2	Poly(rC)-binding protein 2 (Alpha-CP2) (Heterogeneous nuclear ribonucleoprotein E2) (hnRNP E2)	-0.07	9.08E-01	Not Sig.	-0.12	7.84E-01	Not Sig.
Q02952	AKAP12	A-kinase anchor protein 12 (AKAP-12) (A-kinase anchor protein 250 kDa) (AKAP 250) (Gravin) (Myasthenia gravis autoantigen)	-0.04	9.88E-01	Not Sig.	-0.54	5.35E-01	Not Sig.
Q13642	FHL1	Four and a half LIM domains protein 1 (FHL-1) (Skeletal muscle LIM-protein 1) (SLIM) (SLIM-1)	-0.01	9.90E-01	Not Sig.	0.09	8.53E-01	Not Sig.
P08865	RPSA	40S ribosomal protein SA (37 kDa laminin receptor precursor) (37LRP) (37/67 kDa laminin receptor) (LRP/LR) (67 kDa laminin receptor) (67LR) (Colon carcinoma laminin-binding protein) (Laminin receptor 1) (LamR) (Laminin-binding protein precursor p40) (LBP/p40) (Multidrug resistance-associated protein MGr1-Ag) (NEM/1CHD4) (Small ribosomal subunit protein uS2)	-0.47	6.89E-01	Not Sig.	0.22	8.36E-01	Not Sig.
P16401	H1-5	Histone H1.5 (Histone H1a) (Histone H1b) (Histone H1s-3)	0.08	9.60E-01	Not Sig.	1.36	8.89E-02	Not Sig.
P49368	CCT3	T-complex protein 1 subunit gamma (TCP-1-gamma) (CCT-gamma) (hTRiC5)	0.41	4.01E-02	Not Sig.	-0.07	8.23E-01	Not Sig.
P35998	PSMC2	26S proteasome regulatory subunit 7 (26S proteasome AAA-ATPase subunit RPT1) (Proteasome 26S subunit ATPase 2) (Protein MSS1)	-0.27	7.88E-01	Not Sig.	-0.33	7.15E-01	Not Sig.
P35579	MYH9	Myosin-9 (Cellular myosin heavy chain, type A) (Myosin heavy chain 9) (Myosin heavy chain, non-muscle IIa) (Non-muscle myosin heavy chain A) (NMMHC-A) (Non-muscle myosin heavy chain IIa) (NMMHC II-a) (NMMHC-IIA)	-0.34	1.49E-01	Not Sig.	0.04	9.06E-01	Not Sig.
Q99832	CCT7	T-complex protein 1 subunit eta (TCP-1-eta) (CCT-eta) (HIV-1 Nef-interacting protein) [Cleaved into: T-complex protein 1 subunit eta, N-terminally processed]	0.30	2.84E-01	Not Sig.	-0.06	8.55E-01	Not Sig.
Q02790	FKBP4	Peptidyl-prolyl cis-trans isomerase FKBP4 (PPIase FKBP4) (EC 5.2.1.8) (51 kDa	-0.73	3.06E-01	Not Sig.	-0.47	5.17E-01	Not Sig.

		FK506-binding protein) (FKBP51) (52 kDa FK506-binding protein) (52 kDa FKBP) (FKBP-52) (59 kDa immunophilin) (p59) (FK506-binding protein 4) (FKBP-4) (FKBP59) (HSP-binding immunophilin) (HBI) (Immunophilin FKBP52) (Rotamase) [Cleaved into: Peptidyl-prolyl cis-trans isomerase FKBP4, N-terminally processed]						
P19338	NCL	Nucleolin (Protein C23)	0.28	4.77E-01	Not Sig.	0.72	1.66E-02	Not Sig.
Q96QA5	GSDMA	Gasdermin-A (Gasdermin-1) [Cleaved into: Gasdermin-A, N-terminal (GSDMA-NT); Gasdermin-A, C-terminal (GSDMA-CT)]	0.31	8.11E-01	Not Sig.	-0.65	5.37E-01	Not Sig.
Q9Y490	TLN1	Talin-1	-0.06	9.30E-01	Not Sig.	-0.08	8.57E-01	Not Sig.
P26641	EEF1G	Elongation factor 1-gamma (EF-1-gamma) (eEF-1B gamma)	0.00	9.94E-01	Not Sig.	0.01	9.86E-01	Not Sig.
P23381	WARS1	Tryptophan--tRNA ligase, cytoplasmic (EC 6.1.1.2) (Interferon-induced protein 53) (IFP53) (Tryptophanyl-tRNA synthetase) (TrpRS) (hWRS) [Cleaved into: T1-TrpRS; T2-TrpRS]	0.03	9.86E-01	Not Sig.	-0.29	6.79E-01	Not Sig.
P57088	TMEM33	Transmembrane protein 33 (Protein DB83) (SHINC-3)	-0.18	8.63E-01	Not Sig.	-0.58	4.32E-01	Not Sig.
P07900	HSP90AA1	Heat shock protein HSP 90-alpha (EC 3.6.4.10) (Heat shock 86 kDa) (HSP 86) (HSP86) (Lipopolysaccharide-associated protein 2) (LAP-2) (LPS-associated protein 2) (Renal carcinoma antigen NY-REN-38)	-0.22	5.87E-01	Not Sig.	0.03	9.66E-01	Not Sig.
P10809	HSPD1	60 kDa heat shock protein, mitochondrial (EC 5.6.1.7) (60 kDa chaperonin) (Chaperonin 60) (CPN60) (Heat shock protein 60) (HSP-60) (Hsp60) (HuCHA60) (Mitochondrial matrix protein P1) (P60 lymphocyte protein)	0.30	4.15E-01	Not Sig.	0.21	5.49E-01	Not Sig.
P05109	S100A8	Protein S100-A8 (Calgranulin-A) (Calprotectin L1L subunit) (Cystic fibrosis antigen) (CFAG) (Leukocyte L1 complex light chain) (Migration inhibitory factor-related protein 8) (MRP-8) (p8) (S100 calcium-binding protein A8) (Urinary stone protein band A)	-0.40	8.11E-01	Not Sig.	-0.28	8.53E-01	Not Sig.
P27816	MAP4	Microtubule-associated protein 4 (MAP-4)	0.49	5.61E-02	Not Sig.	0.27	3.95E-01	Not Sig.
P40227	CCT6A	T-complex protein 1 subunit zeta (TCP-1-zeta) (Acute morphine dependence-related protein 2) (CCT-zeta-1) (HTR3) (Tcp20)	0.26	5.92E-01	Not Sig.	-0.07	9.06E-01	Not Sig.
P26640	VARS1	Valine--tRNA ligase (EC 6.1.1.9) (Protein G7a) (Valyl-tRNA synthetase) (ValRS)	0.31	7.05E-01	Not Sig.	0.19	8.06E-01	Not Sig.
P04083	ANXA1	Annexin A1 (Annexin I) (Annexin-1) (Calpactin II) (Calpactin-2) (Chromobindin-9) (Lipocortin I) (Phospholipase A2 inhibitory protein) (p35)	-0.47	7.15E-01	Not Sig.	0.24	8.47E-01	Not Sig.
Q86VP6	CAND1	Cullin-associated NEDD8-dissociated protein 1 (Cullin-associated and neddylation-dissociated protein 1) (TBP-interacting protein of 120 kDa A) (TBP-interacting protein 120A) (p120 CAND1)	0.22	7.30E-01	Not Sig.	0.44	3.95E-01	Not Sig.
P04843	RPN1	Dolichyl-diphosphooligosaccharide--protein glycosyltransferase subunit 1 (Dolichyl-diphosphooligosaccharide--protein glycosyltransferase 67 kDa subunit) (Ribophorin I) (RPN-I) (Ribophorin-1)	0.03	9.72E-01	Not Sig.	0.05	9.43E-01	Not Sig.
P07437	TUBB	Tubulin beta chain (Tubulin beta-5 chain)	0.25	4.50E-01	Not Sig.	0.28	3.36E-01	Not Sig.
P55010	EIF5	Eukaryotic translation initiation factor 5 (eIF-5)	-0.44	1.45E-01	Not Sig.	-0.50	8.89E-02	Not Sig.
P54577	YARS1	Tyrosine--tRNA ligase, cytoplasmic (EC 6.1.1.1) (Tyrosyl-tRNA synthetase) (TyrRS) [Cleaved into: Tyrosine--tRNA ligase, cytoplasmic, N-terminally processed]	-0.42	4.81E-01	Not Sig.	0.37	5.12E-01	Not Sig.

P38646	HSPA9	Stress-70 protein, mitochondrial (75 kDa glucose-regulated protein) (GRP-75) (Heat shock 70 kDa protein 9) (Mortalin) (MOT) (Peptide-binding protein 74) (PBP74)	0.11	8.63E-01	Not Sig.	-0.09	8.77E-01	Not Sig.	
Q96RQ3	MCCC1	Methylcrotonoyl-CoA carboxylase subunit alpha, mitochondrial (MCCase subunit alpha) (EC 6.4.1.4) (3-methylcrotonyl-CoA carboxylase 1) (3-methylcrotonyl-CoA carboxylase biotin-containing subunit) (3-methylcrotonyl-CoA:carbon dioxide ligase subunit alpha)	-0.02	9.60E-01	Not Sig.	0.23	3.47E-01	Not Sig.	
P55265	ADAR	Double-stranded RNA-specific adenosine deaminase (DRADA) (EC 3.5.4.37) (136 kDa double-stranded RNA-binding protein) (p136) (Interferon-inducible protein 4) (IFI-4) (K88DSRBP)	-0.41	6.43E-01	Not Sig.	0.09	9.39E-01	Not Sig.	
P46063	RECQL	ATP-dependent DNA helicase Q1 (EC 3.6.4.12) (DNA helicase, RecQ-like type 1) (RecQ1) (DNA-dependent ATPase Q1) (RecQ protein-like 1)	0.02	9.89E-01	Not Sig.	0.15	8.29E-01	Not Sig.	
O00560	SDCBP	Syntenin-1 (Melanoma differentiation-associated protein 9) (MDA-9) (Pro-TGF-alpha cytoplasmic domain-interacting protein 18) (TACIP18) (Scaffold protein Pbp1) (Syndecan-binding protein 1)	-0.82	2.53E-03	Not Sig.	-0.81	4.83E-03	Not Sig.	
P35637	FUS	RNA-binding protein FUS (75 kDa DNA-pairing protein) (Oncogene FUS) (Oncogene TLS) (POMp75) (Translocated in liposarcoma protein)	0.17	8.63E-01	Not Sig.	0.43	5.35E-01	Not Sig.	
P08779	KRT16	Keratin, type I cytoskeletal 16 (Cytokeratin-16) (CK-16) (Keratin-16) (K16)	0.41	8.63E-01	Not Sig.	-1.30	4.29E-01	Not Sig.	
P02533	KRT14	Keratin, type I cytoskeletal 14 (Cytokeratin-14) (CK-14) (Keratin-14) (K14)	0.33	7.97E-01	Not Sig.	-0.63	5.35E-01	Not Sig.	
P01889	HLA-B	HLA class I histocompatibility antigen, B alpha chain (Human leukocyte antigen B) (HLA-B)	-0.24	7.34E-01	Not Sig.	0.05	9.66E-01	Not Sig.	
P35754	GLRX	Glutaredoxin-1 (Thioltransferase-1) (TTase-1)	-0.20	8.63E-01	Not Sig.	-0.08	9.58E-01	Not Sig.	
P04792	HSPB1	Heat shock protein beta-1 (HspB1) (28 kDa heat shock protein) (Estrogen-regulated 24 kDa protein) (Heat shock 27 kDa protein) (HSP 27) (Stress-responsive protein 27) (SRP27)	-0.96	2.50E-03	Not Sig.	-0.25	5.37E-01	Not Sig.	
P26038	MSN	Moesin (Membrane-organizing extension spike protein)	0.28	7.64E-01	Not Sig.	0.27	7.67E-01	Not Sig.	
P18206	VCL	Vinculin (Metavinculin) (MV)	-0.24	8.11E-01	Not Sig.	0.34	7.15E-01	Not Sig.	
O00763	ACACB	Acetyl-CoA carboxylase 2 (EC 6.4.1.2) (ACC-beta)	0.20	4.77E-01	Not Sig.	0.44	4.35E-02	Not Sig.	
Q9NSB4	KRT82	Keratin, type II cuticular Hb2 (Keratin-82) (K82) (Type II hair keratin Hb2) (Type-II keratin Kb22)	-0.35	9.39E-01	Not Sig.	2.52	3.19E-01	Not Sig.	
P13647	KRT5	Keratin, type II cytoskeletal 5 (58 kDa cytokeratin) (Cytokeratin-5) (CK-5) (Keratin-5) (K5) (Type-II keratin Kb5)	0.38	7.16E-01	Not Sig.	-0.36	7.15E-01	Not Sig.	
P62917	RPL8	60S ribosomal protein L8 (Large ribosomal subunit protein uL2)	-0.36	5.30E-01	Not Sig.	-0.18	7.81E-01	Not Sig.	
O15144	ARPC2	Actin-related protein 2/3 complex subunit 2 (Arp2/3 complex 34 kDa subunit) (p34-ARC)	0.52	4.80E-01	Not Sig.	0.11	9.10E-01	Not Sig.	
O15143	ARPC1B	Actin-related protein 2/3 complex subunit 1B (Arp2/3 complex 41 kDa subunit) (p41-ARC)	0.33	7.41E-01	Not Sig.	-0.60	4.51E-01	Not Sig.	
P78344	EIF4G2	Eukaryotic translation initiation factor 4 gamma 2 (eIF-4-gamma 2) (eIF-4G 2) (eIF4G 2) (Death-associated protein 5) (DAP-5) (p97)	-0.42	5.04E-02	Not Sig.	-0.39	8.89E-02	Not Sig.	
P35658	NUP214	Nuclear pore complex protein Nup214 (214 kDa nucleoporin) (Nucleoporin Nup214) (Protein CAN)	-0.15	8.06E-01	Not Sig.	-0.33	4.84E-01	Not Sig.	
P12270	TPR	Nucleoprotein TPR (Megator) (NPC-associated intranuclear protein) (Translocated promoter region protein)	-0.84	6.49E-04	Not Sig.	-0.72	4.83E-03	Not Sig.	

Q96PK6	RBM14	RNA-binding protein 14 (Paraspeckle protein 2) (PSP2) (RNA-binding motif protein 14) (RRM-containing coactivator activator/modulator) (Synaptotagmin-interacting protein) (SYT-interacting protein)	0.22	6.83E-01	Not Sig.	0.29	4.97E-01	Not Sig.
Q16698	DECR1	2,4-dienoyl-CoA reductase [(3E)-enoyl-CoA-producing], mitochondrial (EC 1.3.1.124) (2,4-dienoyl-CoA reductase [NADPH]) (4-enoyl-CoA reductase [NADPH]) (Short chain dehydrogenase/reductase family 18C member 1)	0.08	9.60E-01	Not Sig.	0.28	8.05E-01	Not Sig.
Q06830	PRDX1	Peroxiredoxin-1 (EC 1.11.1.24) (Natural killer cell-enhancing factor A) (NKEF-A) (Proliferation-associated gene protein) (PAG) (Thioredoxin peroxidase 2) (Thioredoxin-dependent peroxide reductase 2) (Thioredoxin-dependent peroxiredoxin 1)	0.30	4.23E-01	Not Sig.	0.13	7.46E-01	Not Sig.
Q5T749	KPRP	Keratinocyte proline-rich protein (hKPRP)	0.47	6.91E-01	Not Sig.	-0.63	5.17E-01	Not Sig.
P04259	KRT6B	Keratin, type II cytoskeletal 6B (Cytokeratin-6B) (CK-6B) (Keratin-6B) (K6B) (Type-II keratin Kb10)	0.15	9.52E-01	Not Sig.	-2.36	5.19E-02	Not Sig.
Q6SA08	TSSK4	Testis-specific serine/threonine-protein kinase 4 (TSK-4) (TSSK-4) (Testis-specific kinase 4) (EC 2.7.11.1) (Serine/threonine-protein kinase 22E)	-0.16	9.27E-01	Not Sig.	0.69	4.94E-01	Not Sig.
Q8N1N4	KRT78	Keratin, type II cytoskeletal 78 (Cytokeratin-78) (CK-78) (Keratin-5b) (Keratin-78) (K78) (Type-II keratin Kb40)	0.57	4.81E-01	Not Sig.	-0.87	1.78E-01	Not Sig.
Q13085	ACACA	Acetyl-CoA carboxylase 1 (ACC1) (EC 6.4.1.2) (Acetyl-Coenzyme A carboxylase alpha) (ACC-alpha)	0.38	4.33E-02	Not Sig.	0.02	9.66E-01	Not Sig.
O00154	ACOT7	Cytosolic acyl coenzyme A thioester hydrolase (EC 3.1.2.2) (Acyl-CoA thioesterase 7) (Brain acyl-CoA hydrolase) (BACH) (hBACH) (CTE-IIa) (CTE-II) (Long chain acyl-CoA thioester hydrolase)	0.28	3.89E-01	Not Sig.	-0.02	9.71E-01	Not Sig.
P60842	EIF4A1	Eukaryotic initiation factor 4A-I (eIF-4A-I) (eIF4A-I) (EC 3.6.4.13) (ATP-dependent RNA helicase eIF4A-1)	0.16	6.07E-01	Not Sig.	0.11	7.15E-01	Not Sig.
Q14574	DSC3	Desmocollin-3 (Cadherin family member 3) (Desmocollin-4) (HT-CP)	0.03	9.90E-01	Not Sig.	-0.44	6.77E-01	Not Sig.
O00487	PSMD14	26S proteasome non-ATPase regulatory subunit 14 (EC 3.4.19.-) (26S proteasome regulatory subunit RPN11) (26S proteasome-associated PAD1 homolog 1)	-0.41	6.33E-01	Not Sig.	-0.25	7.77E-01	Not Sig.
P25705	ATP5F1A	ATP synthase subunit alpha, mitochondrial (ATP synthase F1 subunit alpha)	0.19	7.63E-01	Not Sig.	0.02	9.84E-01	Not Sig.
P06733	ENO1	Alpha-enolase (EC 4.2.1.11) (2-phospho-D-glycerate hydro-lyase) (C-myc promoter-binding protein) (Enolase 1) (MBP-1) (MPB-1) (Non-neural enolase) (NNE) (Phosphopyruvate hydratase) (Plasminogen-binding protein)	0.34	4.15E-01	Not Sig.	0.28	4.88E-01	Not Sig.
P05023	ATP1A1	Sodium/potassium-transporting ATPase subunit alpha-1 (Na(+)/K(+) ATPase alpha-1 subunit) (EC 7.2.2.13) (Sodium pump subunit alpha-1)	-0.86	4.96E-01	Not Sig.	-0.06	9.84E-01	Not Sig.
P23528	CFL1	Cofilin-1 (18 kDa phosphoprotein) (p18) (Cofilin, non-muscle isoform)	-0.06	9.09E-01	Not Sig.	-0.08	8.32E-01	Not Sig.
Q13835	PKP1	Plakophilin-1 (Band 6 protein) (B6P)	0.38	7.43E-01	Not Sig.	0.22	8.36E-01	Not Sig.
P27348	YWHAQ	14-3-3 protein theta (14-3-3 protein T-cell) (14-3-3 protein tau) (Protein HS1)	-0.13	9.10E-01	Not Sig.	0.04	9.84E-01	Not Sig.
P47929	LGALS7	Galectin-7 (Gal-7) (HKL-14) (PI7) (p53-induced gene 1 protein)	-0.82	3.46E-01	Not Sig.	-0.40	6.79E-01	Not Sig.
O75390	CS	Citrate synthase, mitochondrial (EC 2.3.3.1) (Citrate (Si)-synthase)	0.17	7.63E-01	Not Sig.	0.36	4.17E-01	Not Sig.
P50991	CCT4	T-complex protein 1 subunit delta (TCP-1-delta) (CCT-delta) (Stimulator of TAR RNA-binding)	0.24	5.30E-01	Not Sig.	0.06	8.92E-01	Not Sig.
P54136	RARS1	Arginine--tRNA ligase, cytoplasmic (EC 6.1.1.19)	0.00	9.94E-01	Not Sig.	-0.15	7.84E-01	Not Sig.

		(Arginyl-tRNA synthetase) (ArgRS)						
P07814	EPRS1	Bifunctional glutamate/proline--tRNA ligase (Bifunctional aminoacyl-tRNA synthetase) (Cell proliferation-inducing gene 32 protein) (Glutamatyl-prolyl-tRNA synthetase) [Includes: Glutamate--tRNA ligase (EC 6.1.1.17) (Glutamyl-tRNA synthetase) (GluRS); Proline--tRNA ligase (EC 6.1.1.15) (Prolyl-tRNA synthetase)]	0.27	8.11E-01	Not Sig.	-0.39	6.96E-01	Not Sig.
Q99439	CNN2	Calponin-2 (Calponin H2, smooth muscle) (Neutral calponin)	-0.39	7.43E-01	Not Sig.	-0.51	6.29E-01	Not Sig.
P13639	EEF2	Elongation factor 2 (EF-2)	0.01	9.86E-01	Not Sig.	0.14	6.77E-01	Not Sig.
P31949	S100A11	Protein S100-A11 (Calgizzarin) (Metastatic lymph node gene 70 protein) (MLN 70) (Protein S100-C) (S100 calcium-binding protein A11) [Cleaved into: Protein S100-A11, N-terminally processed]	-0.03	9.89E-01	Not Sig.	-0.16	8.76E-01	Not Sig.
P50914	RPL14	60S ribosomal protein L14 (CAG-ISL 7) (Large ribosomal subunit protein eL14)	-0.24	7.45E-01	Not Sig.	0.43	4.88E-01	Not Sig.
Q5T750	XP32	Skin-specific protein 32	0.07	9.72E-01	Not Sig.	-0.22	8.82E-01	Not Sig.
Q14192	FHL2	Four and a half LIM domains protein 2 (FHL-2) (LIM domain protein DRAL) (Skeletal muscle LIM-protein 3) (SLIM-3)	-0.38	4.89E-01	Not Sig.	-0.22	7.15E-01	Not Sig.
P21291	CSRP1	Cysteine and glycine-rich protein 1 (Cysteine-rich protein 1) (CRP) (CRP1) (Epididymis luminal protein 141) (HEL-141)	0.49	4.15E-01	Not Sig.	0.71	1.42E-01	Not Sig.
P07737	PFN1	Profilin-1 (Epididymis tissue protein Li 184a) (Profilin I)	-0.41	1.22E-01	Not Sig.	0.51	5.10E-02	Not Sig.
Q08188	TGM3	Protein-glutamine gamma-glutamyltransferase E (EC 2.3.2.13) (Transglutaminase E) (TG(E)) (TGE) (TGase E) (Transglutaminase-3) (TGase-3) [Cleaved into: Protein-glutamine gamma-glutamyltransferase E 50 kDa catalytic chain; Protein-glutamine gamma-glutamyltransferase E 27 kDa non-catalytic chain]	0.40	7.43E-01	Not Sig.	-0.72	4.83E-01	Not Sig.
Q01130	SRSF2	Serine/arginine-rich splicing factor 2 (Protein PR264) (Splicing component, 35 kDa) (Splicing factor SC35) (SC-35) (Splicing factor, arginine/serine-rich 2)	0.04	9.89E-01	Not Sig.	1.07	1.77E-01	Not Sig.
P11021	HSPA5	Endoplasmic reticulum chaperone BiP (EC 3.6.4.10) (78 kDa glucose-regulated protein) (GRP-78) (Binding-immunoglobulin protein) (BiP) (Heat shock protein 70 family protein 5) (HSP70 family protein 5) (Heat shock protein family A member 5) (Immunoglobulin heavy chain-binding protein)	-0.14	6.83E-01	Not Sig.	0.07	8.23E-01	Not Sig.
P02656	APOC3	Apolipoprotein C-III (Apo-CIII) (ApoC-III) (Apolipoprotein C3)	-0.31	6.96E-01	Not Sig.	0.38	5.49E-01	Not Sig.
Q15717	ELAVL1	ELAV-like protein 1 (Hu-antigen R) (HuR)	0.72	2.05E-01	Not Sig.	-0.45	4.85E-01	Not Sig.
P78371	CCT2	T-complex protein 1 subunit beta (TCP-1-beta) (CCT-beta)	0.09	8.78E-01	Not Sig.	-0.01	9.84E-01	Not Sig.
O95433	AHSA1	Activator of 90 kDa heat shock protein ATPase homolog 1 (AHA1) (p38)	0.30	4.77E-01	Not Sig.	-0.13	7.84E-01	Not Sig.
P81605	DCD	Dermcidin (EC 3.4.-.-) (Preproteolysin) [Cleaved into: Survival-promoting peptide; DCD-1]	-0.10	9.52E-01	Not Sig.	-0.34	7.81E-01	Not Sig.
P18085	ARF4	ADP-ribosylation factor 4	-0.29	7.34E-01	Not Sig.	-0.38	5.83E-01	Not Sig.
P62805	H4C1	Histone H4	-0.89	6.95E-01	Not Sig.	1.00	5.96E-01	Not Sig.
P22234	PAICS	Multifunctional protein ADE2 [Includes: Phosphoribosylaminoimidazole-succinocarboxamide synthase (EC 6.3.2.6) (SAICAR synthetase); Phosphoribosylaminoimidazole carboxylase (EC 4.1.1.21) (AIR carboxylase) (AIRC)]	0.36	6.33E-01	Not Sig.	0.05	9.71E-01	Not Sig.
P37802	TAGLN2	Transgelin-2 (Epididymis tissue protein Li 7e) (SM22-alpha homolog)	-0.80	8.90E-04	Not Sig.	-0.48	6.03E-02	Not Sig.

P84101	SERF2	Small EDRK-rich factor 2 (Gastric cancer-related protein VRG107) (Protein 4F5-related) (4F5rel) (h4F5rel)	-0.49	7.88E-01	Not Sig.	-0.03	9.84E-01	Not Sig.
P53999	SUB1	Activated RNA polymerase II transcriptional coactivator p15 (Positive cofactor 4) (PC4) (SUB1 homolog) (p14)	0.48	7.05E-01	Not Sig.	-0.39	7.33E-01	Not Sig.
P12814	ACTN1	Alpha-actinin-1 (Alpha-actinin cytoskeletal isoform) (F-actin cross-linking protein) (Non-muscle alpha-actinin-1)	0.01	9.94E-01	Not Sig.	-0.02	9.84E-01	Not Sig.
Q13867	BLMH	Bleomycin hydrolase (BH) (BLM hydrolase) (BMH) (EC 3.4.22.40)	0.75	3.78E-01	Not Sig.	-0.45	6.01E-01	Not Sig.
P63244	RACK1	Receptor of activated protein C kinase 1 (Cell proliferation-inducing gene 21 protein) (Guanine nucleotide-binding protein subunit beta-2-like 1) (Guanine nucleotide-binding protein subunit beta-like protein 12.3) (Human lung cancer oncogene 7 protein) (HLC-7) (Receptor for activated C kinase) (Small ribosomal subunit protein RACK1) [Cleaved into: Receptor of activated protein C kinase 1, N-terminally processed (Guanine nucleotide-binding protein subunit beta-2-like 1, N-terminally processed)]	0.37	7.55E-02	Not Sig.	0.07	8.23E-01	Not Sig.
P23284	PPIB	Peptidyl-prolyl cis-trans isomerase B (PPIase B) (EC 5.2.1.8) (CYP-S1) (Cyclophilin B) (Rotamase B) (S-cyclophilin) (SCYLP)	0.37	3.15E-01	Not Sig.	0.45	1.49E-01	Not Sig.
Q9NR12	PDLIM7	PDZ and LIM domain protein 7 (LIM mineralization protein) (LMP) (Protein enigma)	-0.55	5.91E-01	Not Sig.	-1.37	6.64E-02	Not Sig.
Q14204	DYNC1H1	Cytoplasmic dynein 1 heavy chain 1 (Cytoplasmic dynein heavy chain 1) (Dynein heavy chain, cytosolic)	0.19	5.86E-01	Not Sig.	0.22	4.75E-01	Not Sig.
P30041	PRDX6	Peroxiredoxin-6 (EC 1.11.1.27) (1-Cys peroxiredoxin) (1-Cys PRX) (24 kDa protein) (Acidic calcium-independent phospholipase A2) (aiPLA2) (EC 3.1.1.4) (Antioxidant protein 2) (Glutathione-dependent peroxiredoxin) (Liver 2D page spot 40) (Lysophosphatidylcholine acyltransferase 5) (LPC acyltransferase 5) (LPCAT-5) (Lyso-PC acyltransferase 5) (EC 2.3.1.23) (Non-selenium glutathione peroxidase) (NSGPx) (Red blood cells page spot 12)	0.21	6.44E-01	Not Sig.	0.23	5.40E-01	Not Sig.
P30101	PDIA3	Protein disulfide-isomerase A3 (EC 5.3.4.1) (58 kDa glucose-regulated protein) (58 kDa microsomal protein) (p58) (Disulfide isomerase ER-60) (Endoplasmic reticulum resident protein 57) (ER protein 57) (ERp57) (Endoplasmic reticulum resident protein 60) (ER protein 60) (ERp60)	0.26	4.89E-01	Not Sig.	0.41	1.56E-01	Not Sig.
P45974	USP5	Ubiquitin carboxyl-terminal hydrolase 5 (EC 3.4.19.12) (Deubiquitinating enzyme 5) (Isopeptidase T) (Ubiquitin thioesterase 5) (Ubiquitin-specific-processing protease 5)	-0.20	8.35E-01	Not Sig.	-0.26	7.59E-01	Not Sig.
P40939	HADHA	Trifunctional enzyme subunit alpha, mitochondrial (78 kDa gastrin-binding protein) (Monolysocardiolipin acyltransferase) (EC 2.3.1.-) (TP-alpha) [Includes: Long-chain enoyl-CoA hydratase (EC 4.2.1.17); Long chain 3-hydroxyacyl-CoA dehydrogenase (EC 1.1.1.211)]	0.38	2.83E-01	Not Sig.	0.11	8.14E-01	Not Sig.
P08670	VIM	Vimentin	-0.22	5.08E-01	Not Sig.	0.37	1.49E-01	Not Sig.
Q9BY44	EIF2A	Eukaryotic translation initiation factor 2A (eIF-2A) (65 kDa eukaryotic translation initiation factor 2A) [Cleaved into: Eukaryotic translation initiation factor 2A, N-terminally processed]	-0.31	6.89E-01	Not Sig.	0.12	8.61E-01	Not Sig.
P31689	DNAJA1	DnaJ homolog subfamily A member 1 (DnaJ protein	0.12	9.30E-01	Not Sig.	-0.03	9.84E-01	Not Sig.

		homolog 2) (HSDJ) (Heat shock 40 kDa protein 4) (Heat shock protein J2) (HSJ-2) (Human DnaJ protein 2) (hDj-2)						
Q9Y295	DRG1	Developmentally-regulated GTP-binding protein 1 (DRG-1) (Neural precursor cell expressed developmentally down-regulated protein 3) (NEDD-3) (Translation factor GTPase DRG1) (TRAFAC GTPase DRG1) (EC 3.6.5.-)	-0.82	6.29E-02	Not Sig.	-0.44	4.26E-01	Not Sig.
P35527	KRT9	Keratin, type I cytoskeletal 9 (Cytokeratin-9) (CK-9) (Keratin-9) (K9)	0.68	2.24E-01	Not Sig.	-0.17	8.17E-01	Not Sig.
P46777	RPL5	60S ribosomal protein L5 (Large ribosomal subunit protein uL18)	0.10	8.63E-01	Not Sig.	0.58	1.29E-01	Not Sig.
P62249	RPS16	40S ribosomal protein S16 (Small ribosomal subunit protein uS9)	0.11	7.97E-01	Not Sig.	0.05	9.27E-01	Not Sig.
P62753	RPS6	40S ribosomal protein S6 (Phosphoprotein NP33) (Small ribosomal subunit protein eS6)	-0.16	7.88E-01	Not Sig.	0.51	1.92E-01	Not Sig.
Q14764	MVP	Major vault protein (MVP) (Lung resistance-related protein)	-0.61	3.32E-01	Not Sig.	-0.90	8.89E-02	Not Sig.
P16615	ATP2A2	Sarcoplasmic/endoplasmic reticulum calcium ATPase 2 (SERCA2) (SR Ca(2+)-ATPase 2) (EC 7.2.2.10) (Calcium pump 2) (Calcium-transporting ATPase sarcoplasmic reticulum type, slow twitch skeletal muscle isoform) (Endoplasmic reticulum class 1/2 Ca(2+) ATPase)	0.23	7.34E-01	Not Sig.	-0.17	7.84E-01	Not Sig.
Q99829	CPNE1	Copine-1 (Chromobindin 17) (Copine I)	-0.41	5.65E-01	Not Sig.	0.18	8.18E-01	Not Sig.
P13646	KRT13	Keratin, type I cytoskeletal 13 (Cytokeratin-13) (CK-13) (Keratin-13) (K13)	-0.31	9.18E-01	Not Sig.	-0.11	9.80E-01	Not Sig.
P00338	LDHA	L-lactate dehydrogenase A chain (LDH-A) (EC 1.1.1.27) (Cell proliferation-inducing gene 19 protein) (LDH muscle subunit) (LDH-M) (Renal carcinoma antigen NY-REN-59)	0.34	1.48E-01	Not Sig.	0.36	1.03E-01	Not Sig.
P61978	HNRNPK	Heterogeneous nuclear ribonucleoprotein K (hnRNP K) (Transformation up-regulated nuclear protein) (TUNP)	-0.32	4.15E-01	Not Sig.	-0.24	5.35E-01	Not Sig.
Q14498	RBM39	RNA-binding protein 39 (CAPER alpha) (CAPERalpha) (Hepatocellular carcinoma protein 1) (RNA-binding motif protein 39) (RNA-binding region-containing protein 2) (Splicing factor HCC1)	0.36	6.04E-01	Not Sig.	0.25	7.15E-01	Not Sig.
P55060	CSE1L	Exportin-2 (Exp2) (Cellular apoptosis susceptibility protein) (Chromosome segregation 1-like protein) (Importin-alpha re-exporter)	0.30	5.70E-01	Not Sig.	0.02	9.84E-01	Not Sig.
O60506	SYNCRIP	Heterogeneous nuclear ribonucleoprotein Q (hnRNP Q) (Glycine- and tyrosine-rich RNA-binding protein) (GRY-RBP) (NS1-associated protein 1) (Synaptotagmin-binding, cytoplasmic RNA-interacting protein)	-0.19	8.11E-01	Not Sig.	0.10	9.06E-01	Not Sig.
P04040	CAT	Catalase (EC 1.11.1.6)	0.95	4.15E-01	Not Sig.	-0.01	9.94E-01	Not Sig.
Q92616	GCN1	eIF-2-alpha kinase activator GCN1 (GCN1 eIF-2-alpha kinase activator homolog) (GCN1-like protein 1) (General control of amino-acid synthesis 1-like protein 1) (Translational activator GCN1) (HsGCN1)	-0.38	5.31E-01	Not Sig.	0.09	9.10E-01	Not Sig.
Q13546	RIPK1	Receptor-interacting serine/threonine-protein kinase 1 (EC 2.7.11.1) (Cell death protein RIP) (Receptor-interacting protein 1) (RIP-1)	-0.85	6.96E-02	Not Sig.	-0.41	4.88E-01	Not Sig.
P61160	ACTR2	Actin-related protein 2 (Actin-like protein 2)	0.24	6.32E-01	Not Sig.	-0.03	9.66E-01	Not Sig.
Q00839	HNRNPU	Heterogeneous nuclear ribonucleoprotein U (hnRNP U) (GRIP120) (Nuclear p120 ribonucleoprotein) (Scaffold-attachment factor A) (SAF-A) (p120) (pp120)	-0.05	9.52E-01	Not Sig.	0.30	4.94E-01	Not Sig.
Q99623	PHB2	Prohibitin-2 (B-cell receptor-associated protein BAP37) (D-	-0.62	1.69E-01	Not Sig.	-0.75	8.25E-02	Not Sig.

		prohibitin) (Repressor of estrogen receptor activity)						
P06702	S100A9	Protein S100-A9 (Calgranulin-B) (Calprotectin L1H subunit) (Leukocyte L1 complex heavy chain) (Migration inhibitory factor-related protein 14) (MRP-14) (p14) (S100 calcium-binding protein A9)	-0.55	6.87E-01	Not Sig.	-0.19	8.93E-01	Not Sig.
P12004	PCNA	Proliferating cell nuclear antigen (PCNA) (Cyclin)	-0.10	9.30E-01	Not Sig.	-0.37	5.96E-01	Not Sig.
P21980	TGM2	Protein-glutamine gamma-glutamyltransferase 2 (EC 2.3.2.13) (Erythrocyte transglutaminase) (Heart G alpha(h)) (hhG alpha(h)) (Isopeptidase TGM2) (EC 3.4.-.-) (Protein G alpha(h)) (G(h)) (Protein-glutamine deamidase TGM2) (EC 3.5.1.44) (Protein-glutamine dopaminyltransferase TGM2) (EC 2.3.1.-) (Protein-glutamine histaminyltransferase TGM2) (EC 2.3.1.-) (Protein-glutamine noradrenalinyltransferase TGM2) (EC 2.3.1.-) (Protein-glutamine serotonyltransferase TGM2) (EC 2.3.1.-) (Tissue transglutaminase) (tTG) (tTgase) (Transglutaminase C) (TG(C)) (TGC) (TGase C) (Transglutaminase H) (TGase H) (Transglutaminase II) (TGase II) (Transglutaminase-2) (TG2) (TGase-2) (hTG2)	0.46	6.31E-01	Not Sig.	0.09	9.43E-01	Not Sig.
Q92841	DDX17	Probable ATP-dependent RNA helicase DDX17 (EC 3.6.4.13) (DEAD box protein 17) (DEAD box protein p72) (DEAD box protein p82) (RNA-dependent helicase p72)	0.30	6.65E-01	Not Sig.	-0.15	8.23E-01	Not Sig.
P31944	CASP14	Caspase-14 (CASP-14) (EC 3.4.22.-) [Cleaved into: Caspase-14 subunit p17, mature form; Caspase-14 subunit p10, mature form; Caspase-14 subunit p20, intermediate form; Caspase-14 subunit p8, intermediate form]	0.84	6.04E-01	Not Sig.	-0.88	5.35E-01	Not Sig.
P55084	HADHB	Trifunctional enzyme subunit beta, mitochondrial (TP-beta) [Includes: 3-ketoacyl-CoA thiolase (EC 2.3.1.155) (EC 2.3.1.16) (Acetyl-CoA acyltransferase) (Beta-ketothiolase)]	0.52	3.89E-01	Not Sig.	-0.23	7.33E-01	Not Sig.
Q9NZI8	IGF2BP1	Insulin-like growth factor 2 mRNA-binding protein 1 (IGF2 mRNA-binding protein 1) (IMP-1) (IMP1) (Coding region determinant-binding protein) (CRD-BP) (IGF-II mRNA-binding protein 1) (VICKZ family member 1) (Zipcode-binding protein 1) (ZBP-1)	-0.07	9.52E-01	Not Sig.	0.26	7.31E-01	Not Sig.
P62277	RPS13	40S ribosomal protein S13 (Small ribosomal subunit protein uS15)	-0.09	9.51E-01	Not Sig.	0.49	5.12E-01	Not Sig.
P35030	PRSS3	Trypsin-3 (EC 3.4.21.4) (Brain trypsinogen) (Mesotrypsin) (Mesotrypsinogen) (Serine protease 3) (Serine protease 4) (Trypsin III) (Trypsin IV)	0.00	9.94E-01	Not Sig.	0.10	7.99E-01	Not Sig.
P18754	RCC1	Regulator of chromosome condensation (Cell cycle regulatory protein) (Chromosome condensation protein 1)	0.07	9.27E-01	Not Sig.	0.22	6.39E-01	Not Sig.
Q3SY84	KRT71	Keratin, type II cytoskeletal 71 (Cytokeratin-71) (CK-71) (Keratin-71) (K71) (Type II inner root sheath-specific keratin-K6irs1) (Keratin 6 irs) (hK6irs) (hK6irs1) (Type-II keratin Kb34)	0.01	9.90E-01	Not Sig.	0.41	5.49E-01	Not Sig.
P48643	CCT5	T-complex protein 1 subunit epsilon (TCP-1-epsilon) (CCT-epsilon)	-0.05	9.30E-01	Not Sig.	-0.01	9.84E-01	Not Sig.
P35908	KRT2	Keratin, type II cytoskeletal 2 epidermal (Cytokeratin-2e) (CK-2e) (Epithelial keratin-2e) (Keratin-2 epidermis) (Keratin-2e) (K2e) (Type-II keratin Kb2)	0.49	6.31E-01	Not Sig.	-0.56	5.19E-01	Not Sig.
P05089	ARG1	Arginase-1 (EC 3.5.3.1) (Liver-type arginase) (Type I arginase)	0.96	2.48E-01	Not Sig.	-0.69	4.32E-01	Not Sig.

P36952	SERPINB5	Serpin B5 (Maspin) (Peptidase inhibitor 5) (PI-5)	0.24	7.64E-01	Not Sig.	-0.14	8.53E-01	Not Sig.
P38919	EIF4A3	Eukaryotic initiation factor 4A-III (eIF-4A-III) (eIF4A-III) (EC 3.6.4.13) (ATP-dependent RNA helicase DDX48) (ATP-dependent RNA helicase eIF4A-3) (DEAD box protein 48) (Eukaryotic initiation factor 4A-like NUK-34) (Eukaryotic translation initiation factor 4A isoform 3) (Nuclear matrix protein 265) (NMP 265) (hNMP 265) [Cleaved into: Eukaryotic initiation factor 4A-III, N-terminally processed]	-0.10	9.30E-01	Not Sig.	0.24	7.86E-01	Not Sig.
Q15084	PDIA6	Protein disulfide-isomerase A6 (EC 5.3.4.1) (Endoplasmic reticulum protein 5) (ER protein 5) (ERp5) (Protein disulfide isomerase P5) (Thioredoxin domain-containing protein 7)	0.29	6.32E-01	Not Sig.	0.01	9.86E-01	Not Sig.
P17844	DDX5	Probable ATP-dependent RNA helicase DDX5 (EC 3.6.4.13) (DEAD box protein 5) (RNA helicase p68)	0.06	8.74E-01	Not Sig.	-0.08	8.14E-01	Not Sig.
P26599	PTBP1	Polypyrimidine tract-binding protein 1 (PTB) (57 kDa RNA-binding protein PPTB-1) (Heterogeneous nuclear ribonucleoprotein I) (hnRNP I)	-0.12	7.97E-01	Not Sig.	-0.03	9.71E-01	Not Sig.
P62280	RPS11	40S ribosomal protein S11 (Small ribosomal subunit protein uS17)	-0.48	4.77E-01	Not Sig.	0.11	8.89E-01	Not Sig.
P47756	CAPZB	F-actin-capping protein subunit beta (CapZ beta)	0.40	3.78E-01	Not Sig.	-0.20	6.77E-01	Not Sig.
O75083	WDR1	WD repeat-containing protein 1 (Actin-interacting protein 1) (AIP1) (NORI-1)	-0.23	6.31E-01	Not Sig.	-0.02	9.84E-01	Not Sig.
P26373	RPL13	60S ribosomal protein L13 (Breast basic conserved protein 1) (Large ribosomal subunit protein eL13)	0.07	8.63E-01	Not Sig.	0.12	7.33E-01	Not Sig.
P60660	MYL6	Myosin light polypeptide 6 (17 kDa myosin light chain) (LC17) (Myosin light chain 3) (MLC-3) (Myosin light chain alkali 3) (Myosin light chain A3) (Smooth muscle and nonmuscle myosin light chain alkali 6)	-0.33	7.43E-01	Not Sig.	-0.29	7.46E-01	Not Sig.
Q8NC51	SERBP1	Plasminogen activator inhibitor 1 RNA-binding protein (PAI1 RNA-binding protein 1) (PAI-RBP1) (SERPINE1 mRNA-binding protein 1)	-0.26	7.63E-01	Not Sig.	-0.40	5.73E-01	Not Sig.
O43707	ACTN4	Alpha-actinin-4 (Non-muscle alpha-actinin 4)	0.15	8.63E-01	Not Sig.	0.71	1.57E-01	Not Sig.
Q14008	CKAP5	Cytoskeleton-associated protein 5 (Colonic and hepatic tumor overexpressed gene protein) (Ch-TOG)	-0.11	8.66E-01	Not Sig.	0.01	9.84E-01	Not Sig.
P11308	ERG	Transcriptional regulator ERG (Transforming protein ERG)	0.23	8.63E-01	Not Sig.	0.31	7.96E-01	Not Sig.
P49321	NASP	Nuclear autoantigenic sperm protein (NASP)	0.16	8.19E-01	Not Sig.	-0.12	8.55E-01	Not Sig.
P27708	CAD	CAD protein [Includes: Glutamine-dependent carbamoyl-phosphate synthase (EC 6.3.5.5); Aspartate carbamoyltransferase (EC 2.1.3.2); Dihydroorotase (EC 3.5.2.3)]	0.71	1.20E-01	Not Sig.	-0.16	8.05E-01	Not Sig.
Q04637	EIF4G1	Eukaryotic translation initiation factor 4 gamma 1 (eIF-4-gamma 1) (eIF-4G 1) (eIF-4G1) (p220)	0.04	9.60E-01	Not Sig.	0.13	7.84E-01	Not Sig.
P62701	RPS4X	40S ribosomal protein S4, X isoform (SCR10) (Single copy abundant mRNA protein) (Small ribosomal subunit protein eS4)	0.16	6.70E-01	Not Sig.	-0.03	9.58E-01	Not Sig.
Q99798	ACO2	Aconitate hydratase, mitochondrial (Aconitase) (EC 4.2.1.3) (Citrate hydro-lyase)	0.16	8.41E-01	Not Sig.	0.30	6.43E-01	Not Sig.
Q13151	HNRNPA0	Heterogeneous nuclear ribonucleoprotein A0 (hnRNP A0)	0.32	4.50E-01	Not Sig.	0.48	1.49E-01	Not Sig.
P63241	EIF5A	Eukaryotic translation initiation factor 5A-1 (eIF-5A-1) (eIF-5A1) (Eukaryotic initiation factor 5A isoform 1) (eIF-5A) (Rev-binding factor) (eIF-4D)	0.33	2.31E-01	Not Sig.	0.35	1.49E-01	Not Sig.
P46940	IQGAP1	Ras GTPase-activating-like protein IQGAP1 (p195)	-0.43	5.61E-01	Not Sig.	0.32	6.62E-01	Not Sig.
P51991	HNRNPA3	Heterogeneous nuclear ribonucleoprotein A3 (hnRNP A3)	-0.09	8.90E-01	Not Sig.	0.14	7.84E-01	Not Sig.

Q9Y3U8	RPL36	60S ribosomal protein L36 (Large ribosomal subunit protein eL36)	-0.23	6.87E-01	Not Sig.	-0.03	9.71E-01	Not Sig.
P22626	HNRNPA 2B1	Heterogeneous nuclear ribonucleoproteins A2/B1 (hnRNP A2/B1)	0.06	9.60E-01	Not Sig.	0.37	5.91E-01	Not Sig.
P62857	RPS28	40S ribosomal protein S28 (Small ribosomal subunit protein eS28)	-0.12	9.09E-01	Not Sig.	0.00	9.96E-01	Not Sig.
P32119	PRDX2	Peroxiredoxin-2 (EC 1.11.1.24) (Natural killer cell-enhancing factor B) (NKEF-B) (PRP) (Thiol-specific antioxidant protein) (TSA) (Thioredoxin peroxidase 1) (Thioredoxin-dependent peroxide reductase 1) (Thioredoxin-dependent peroxiredoxin 2)	0.82	4.15E-01	Not Sig.	-0.41	7.15E-01	Not Sig.
P62937	PPIA	Peptidyl-prolyl cis-trans isomerase A (PPIase A) (EC 5.2.1.8) (Cyclophilin A) (Cyclosporin A-binding protein) (Rotamase A) [Cleaved into: Peptidyl-prolyl cis-trans isomerase A, N-terminally processed]	0.25	4.77E-01	Not Sig.	0.45	9.10E-02	Not Sig.
P61927	RPL37	60S ribosomal protein L37 (G1.16) (Large ribosomal subunit protein eL37)	-0.17	8.92E-01	Not Sig.	0.03	9.84E-01	Not Sig.
O75534	CSDE1	Cold shock domain-containing protein E1 (N-ras upstream gene protein) (Protein UNR)	-0.67	2.25E-01	Not Sig.	-0.83	8.89E-02	Not Sig.
Q9HB7 1	CACYBP	Calcyclin-binding protein (CacyBP) (hCacyBP) (S100A6-binding protein) (Siah-interacting protein)	-0.60	1.17E-02	Not Sig.	-0.97	1.87E-04	Not Sig.
P49915	GMPS	GMP synthase [glutamine-hydrolyzing] (EC 6.3.5.2) (GMP synthetase) (Glutamine amidotransferase)	-0.02	9.86E-01	Not Sig.	-0.20	7.41E-01	Not Sig.
Q14980	NUMA1	Nuclear mitotic apparatus protein 1 (Nuclear matrix protein-22) (NMP-22) (Nuclear mitotic apparatus protein) (NuMA protein) (SP-H antigen)	-0.58	4.96E-01	Not Sig.	0.71	3.65E-01	Not Sig.
Q01469	FABP5	Fatty acid-binding protein 5 (Epidermal-type fatty acid-binding protein) (E-FABP) (Fatty acid-binding protein, epidermal) (Psoriasis-associated fatty acid-binding protein homolog) (PA-FABP)	0.13	9.52E-01	Not Sig.	-0.26	8.55E-01	Not Sig.
Q5D862	FLG2	Filaggrin-2 (FLG-2) (Intermediate filament-associated and psoriasis-susceptibility protein) (Ifapsoriasin)	0.34	7.43E-01	Not Sig.	-1.16	8.89E-02	Not Sig.
P46781	RPS9	40S ribosomal protein S9 (Small ribosomal subunit protein uS4)	0.23	4.78E-01	Not Sig.	0.24	4.18E-01	Not Sig.
P26368	U2AF2	Splicing factor U2AF 65 kDa subunit (U2 auxiliary factor 65 kDa subunit) (hU2AF(65)) (hU2AF65) (U2 snRNP auxiliary factor large subunit)	-0.26	7.62E-01	Not Sig.	0.74	1.92E-01	Not Sig.
Q00341	HDLBP	Vigilin (High density lipoprotein-binding protein) (HDL-binding protein)	-0.10	9.03E-01	Not Sig.	0.39	4.34E-01	Not Sig.
P04075	ALDOA	Fructose-bisphosphate aldolase A (EC 4.1.2.13) (Lung cancer antigen NY-LU-1) (Muscle-type aldolase)	0.00	9.94E-01	Not Sig.	-0.52	2.15E-01	Not Sig.
P15311	EZR	Ezrin (Cytovillin) (Villin-2) (p81)	-0.45	3.55E-01	Not Sig.	0.49	2.31E-01	Not Sig.
Q03181	PPARD	Peroxisome proliferator-activated receptor delta (PPAR-delta) (NUCI) (Nuclear hormone receptor 1) (NUC1) (Nuclear receptor subfamily 1 group C member 2) (Peroxisome proliferator-activated receptor beta) (PPAR-beta)	-0.47	7.34E-01	Not Sig.	0.04	9.84E-01	Not Sig.
Q01813	PFKP	ATP-dependent 6-phosphofructokinase, platelet type (ATP-PFK) (PFK-P) (EC 2.7.1.11) (6-phosphofructokinase type C) (Phosphofructo-1-kinase isozyme C) (PFK-C) (Phosphohexokinase)	-0.10	8.63E-01	Not Sig.	0.17	7.41E-01	Not Sig.
P08243	ASNS	Asparagine synthetase [glutamine-hydrolyzing] (EC 6.3.5.4) (Cell cycle control protein TS11) (Glutamine-dependent asparagine synthetase)	-0.55	5.52E-01	Not Sig.	-0.59	4.83E-01	Not Sig.
Q02413	DSG1	Desmoglein-1 (Cadherin family member 4) (Desmosomal glycoprotein 1) (DG1) (DGI) (Pemphigus foliaceus antigen)	0.83	3.15E-01	Not Sig.	-0.40	6.77E-01	Not Sig.

P55072	VCP	Transitional endoplasmic reticulum ATPase (TER ATPase) (EC 3.6.4.6) (15S Mg(2+)-ATPase p97 subunit) (Valosin-containing protein) (VCP)	-0.94	8.56E-02	Not Sig.	0.43	5.35E-01	Not Sig.
Q8IZH2	XRN1	5'-3' exoribonuclease 1 (EC 3.1.13.-) (Strand-exchange protein 1 homolog)	-0.77	5.01E-02	Not Sig.	-0.59	1.52E-01	Not Sig.
P20930	FLG	Filaggrin	0.94	3.13E-01	Not Sig.	-0.07	9.71E-01	Not Sig.
P46778	RPL21	60S ribosomal protein L21 (Large ribosomal subunit protein eL21)	0.46	5.81E-01	Not Sig.	-0.31	7.15E-01	Not Sig.
Q13200	PSMD2	26S proteasome non-ATPase regulatory subunit 2 (26S proteasome regulatory subunit RPN1) (26S proteasome regulatory subunit S2) (26S proteasome subunit p97) (Protein 55.11) (Tumor necrosis factor type 1 receptor-associated protein 2)	-0.22	7.05E-01	Not Sig.	0.13	8.23E-01	Not Sig.
O15427	SLC16A3	Monocarboxylate transporter 4 (MCT 4) (Solute carrier family 16 member 3)	0.51	3.04E-01	Not Sig.	0.21	7.15E-01	Not Sig.
Q08378	GOLGA3	Golgin subfamily A member 3 (Golgi complex-associated protein of 170 kDa) (GCP170) (Golgin-160)	-0.53	4.15E-01	Not Sig.	0.09	9.14E-01	Not Sig.
P18621	RPL17	60S ribosomal protein L17 (60S ribosomal protein L23) (Large ribosomal subunit protein uL22) (PD-1)	0.15	8.63E-01	Not Sig.	-0.29	6.69E-01	Not Sig.
Q6P2Q9	PRPF8	Pre-mRNA-processing-splicing factor 8 (220 kDa U5 snRNP-specific protein) (PRP8 homolog) (Splicing factor Prp8) (p220)	-0.41	5.03E-01	Not Sig.	-0.23	7.24E-01	Not Sig.
P11586	MTHFD1	C-1-tetrahydrofolate synthase, cytoplasmic (C1-THF synthase) (Epididymis secretory sperm binding protein) [Cleaved into: C-1-tetrahydrofolate synthase, cytoplasmic, N-terminally processed] [Includes: Methylenetetrahydrofolate dehydrogenase (EC 1.5.1.5); Methenyltetrahydrofolate cyclohydrolase (EC 3.5.4.9); Formyltetrahydrofolate synthetase (EC 6.3.4.3)]	0.60	2.75E-02	Not Sig.	0.21	5.49E-01	Not Sig.
Q6IBS0	TWF2	Twinfilin-2 (A6-related protein) (hA6RP) (Protein tyrosine kinase 9-like) (Twinfilin-1-like protein)	-0.41	7.09E-01	Not Sig.	-0.66	4.78E-01	Not Sig.
O43143	DHX15	Pre-mRNA-splicing factor ATP-dependent RNA helicase DHX15 (EC 3.6.4.13) (ATP-dependent RNA helicase #46) (DEAH box protein 15)	0.24	7.54E-01	Not Sig.	0.14	8.50E-01	Not Sig.
Q8WUM4	PDCD6IP	Programmed cell death 6-interacting protein (PDCD6-interacting protein) (ALG-2-interacting protein 1) (ALG-2-interacting protein X) (Hp95)	0.07	9.30E-01	Not Sig.	0.42	3.95E-01	Not Sig.
P42357	HAL	Histidine ammonia-lyase (Histidase) (EC 4.3.1.3)	0.60	6.65E-01	Not Sig.	-0.84	4.69E-01	Not Sig.
Q16658	FSCN1	Fascin (55 kDa actin-bundling protein) (Singed-like protein) (p55)	0.35	3.13E-01	Not Sig.	0.17	6.58E-01	Not Sig.
Q9P2J5	LARS1	Leucine--tRNA ligase, cytoplasmic (EC 6.1.1.4) (Leucyl-tRNA synthetase) (LeuRS)	-0.48	4.50E-01	Not Sig.	-0.13	8.53E-01	Not Sig.
O00159	MYO1C	Unconventional myosin-Ic (Myosin I beta) (MMI-beta) (MMIb)	0.39	3.13E-01	Not Sig.	-0.04	9.58E-01	Not Sig.
Q8WWM7	ATXN2L	Ataxin-2-like protein (Ataxin-2 domain protein) (Ataxin-2-related protein)	0.31	7.43E-01	Not Sig.	0.44	5.49E-01	Not Sig.
Q14103	HNRNPD	Heterogeneous nuclear ribonucleoprotein D0 (hnRNP D0) (AU-rich element RNA-binding protein 1)	-0.05	9.52E-01	Not Sig.	0.25	6.36E-01	Not Sig.
Q00325	SLC25A3	Phosphate carrier protein, mitochondrial (Phosphate transport protein) (PTP) (Solute carrier family 25 member 3)	0.23	6.31E-01	Not Sig.	-0.04	9.50E-01	Not Sig.
Q6UWP8	SBSN	Suprabasin	0.10	9.30E-01	Not Sig.	-0.55	4.04E-01	Not Sig.
P07195	LDHB	L-lactate dehydrogenase B chain (LDH-B) (EC 1.1.1.27) (LDH heart subunit) (LDH-H) (Renal carcinoma antigen NY-REN-46)	0.20	6.44E-01	Not Sig.	0.24	5.17E-01	Not Sig.
P35237	SERPINB6	Serpin B6 (Cytoplasmic antiproteinase) (CAP) (Peptidase inhibitor 6) (PI-6) (Placental thrombin inhibitor)	-0.01	9.90E-01	Not Sig.	0.02	9.84E-01	Not Sig.

P32969	RPL9	60S ribosomal protein L9 (Large ribosomal subunit protein uL6)	-0.20	8.09E-01	Not Sig.	-0.37	5.60E-01	Not Sig.
P41252	IARS1	Isoleucine--tRNA ligase, cytoplasmic (EC 6.1.1.5) (Isoleucyl-tRNA synthetase) (IRS) (IleRS)	0.11	9.30E-01	Not Sig.	0.06	9.66E-01	Not Sig.
O75342	ALOX12B	Arachidonate 12-lipoxygenase, 12R-type (12R-LOX) (12R-lipoxygenase) (EC 1.13.11.-) (Epidermis-type lipoxygenase 12)	0.01	9.94E-01	Not Sig.	-0.31	7.73E-01	Not Sig.
P61158	ACTR3	Actin-related protein 3 (Actin-like protein 3)	-0.27	7.34E-01	Not Sig.	0.07	9.48E-01	Not Sig.
P27635	RPL10	60S ribosomal protein L10 (Laminin receptor homolog) (Large ribosomal subunit protein uL16) (Protein QM) (Ribosomal protein L10) (Tumor suppressor QM)	-0.58	1.97E-02	Not Sig.	-0.43	1.05E-01	Not Sig.
P62826	RAN	GTP-binding nuclear protein Ran (Androgen receptor-associated protein 24) (GTPase Ran) (Ras-like protein TC4) (Ras-related nuclear protein)	0.32	3.68E-01	Not Sig.	0.22	5.32E-01	Not Sig.
P01857	IGHG1	Immunoglobulin heavy constant gamma 1 (Ig gamma-1 chain C region) (Ig gamma-1 chain C region EU) (Ig gamma-1 chain C region KOL) (Ig gamma-1 chain C region NIE)	0.31	8.25E-01	Not Sig.	0.51	6.77E-01	Not Sig.
Q9UBC9	SPRR3	Small proline-rich protein 3 (22 kDa pancornulin) (Cornifin beta) (Esophagin)	-0.37	8.30E-01	Not Sig.	0.78	5.49E-01	Not Sig.
P30044	PRDX5	Peroxiredoxin-5, mitochondrial (EC 1.11.1.24) (Alu corepressor 1) (Antioxidant enzyme B166) (AOEB166) (Liver tissue 2D-page spot 71B) (PLP) (Peroxiredoxin V) (Prx-V) (Peroxisomal antioxidant enzyme) (TPx type VI) (Thioredoxin peroxidase PMP20) (Thioredoxin-dependent peroxiredoxin 5)	-0.51	3.85E-01	Not Sig.	-0.23	7.31E-01	Not Sig.
P52907	CAPZA1	F-actin-capping protein subunit alpha-1 (CapZ alpha-1)	-0.19	7.97E-01	Not Sig.	-0.14	8.32E-01	Not Sig.
P06576	ATP5F1B	ATP synthase subunit beta, mitochondrial (EC 7.1.2.2) (ATP synthase F1 subunit beta)	-0.40	7.64E-01	Not Sig.	0.29	8.23E-01	Not Sig.
P17812	CTPS1	CTP synthase 1 (EC 6.3.4.2) (CTP synthetase 1) (UTP--ammonia ligase 1)	0.12	8.11E-01	Not Sig.	0.22	6.31E-01	Not Sig.
P62861	FAU	40S ribosomal protein S30 (Small ribosomal subunit protein eS30)	-0.18	8.63E-01	Not Sig.	1.09	7.45E-02	Not Sig.
O96008	TOMM40	Mitochondrial import receptor subunit TOM40 homolog (Protein Haymaker) (Translocase of outer membrane 40 kDa subunit homolog) (p38.5)	-0.02	9.89E-01	Not Sig.	0.14	8.53E-01	Not Sig.
P50395	GDI2	Rab GDP dissociation inhibitor beta (Rab GDI beta) (Guanosine diphosphate dissociation inhibitor 2) (GDI-2)	-0.19	8.03E-01	Not Sig.	0.22	7.33E-01	Not Sig.
P12273	PIP	Prolactin-inducible protein (Gross cystic disease fluid protein 15) (GCDFP-15) (Prolactin-induced protein) (Secretory actin-binding protein) (SABP) (gp17)	0.08	9.60E-01	Not Sig.	-0.26	8.23E-01	Not Sig.
P58546	MTPN	Myotrophin (Protein V-1)	-0.11	8.63E-01	Not Sig.	-0.73	8.25E-02	Not Sig.
Q08J23	NSUN2	RNA cytosine C(5)-methyltransferase NSUN2 (EC 2.1.1.-) (Myc-induced SUN domain-containing protein) (Misu) (NOL1/NOP2/Sun domain family member 2) (Substrate of AIM1/Aurora kinase B) (mRNA cytosine C(5)-methyltransferase) (EC 2.1.1.-) (tRNA cytosine C(5)-methyltransferase) (EC 2.1.1.-) (EC 2.1.1.203) (tRNA methyltransferase 4 homolog) (hTrm4)	-0.13	8.63E-01	Not Sig.	-0.38	5.06E-01	Not Sig.
Q9P2K5	MYEF2	Myelin expression factor 2 (MEF-2) (MyEF-2) (MST156)	-0.10	9.30E-01	Not Sig.	0.49	4.85E-01	Not Sig.
Q8NE71	ABCF1	ATP-binding cassette sub-family F member 1 (ATP-binding cassette 50) (TNF-alpha-stimulated ABC protein)	0.30	7.63E-01	Not Sig.	-0.34	7.15E-01	Not Sig.
P40926	MDH2	Malate dehydrogenase, mitochondrial (EC 1.1.1.37)	-0.03	9.86E-01	Not Sig.	0.09	9.31E-01	Not Sig.
Q07020	RPL18	60S ribosomal protein L18 (Large ribosomal subunit protein eL18)	-0.06	9.44E-01	Not Sig.	0.21	6.77E-01	Not Sig.
P43243	MATR3	Matrin-3	-0.14	8.63E-01	Not Sig.	0.10	9.06E-01	Not Sig.

Q8NFW1	COL22A1	Collagen alpha-1(XXII) chain	-0.58	6.65E-01	Not Sig.	0.03	9.84E-01	Not Sig.
Q14157	UBAP2L	Ubiquitin-associated protein 2-like (Protein NICE-4)	-0.45	4.47E-01	Not Sig.	-0.60	1.99E-01	Not Sig.
P45880	VDAC2	Voltage-dependent anion-selective channel protein 2 (VDAC-2) (hVDAC2) (Outer mitochondrial membrane protein porin 2)	0.02	9.72E-01	Not Sig.	0.09	7.96E-01	Not Sig.
P62495	ETF1	Eukaryotic peptide chain release factor subunit 1 (Eukaryotic release factor 1) (eRF1) (Protein Cl1) (TB3-1)	-0.18	8.63E-01	Not Sig.	0.74	3.36E-01	Not Sig.
Q9NTK5	OLA1	Obg-like ATPase 1 (DNA damage-regulated overexpressed in cancer 45) (DOC45) (GTP-binding protein 9)	-0.14	9.30E-01	Not Sig.	-0.42	6.77E-01	Not Sig.
Q9NZ08	ERAP1	Endoplasmic reticulum aminopeptidase 1 (EC 3.4.11.-) (ARTS-1) (Adipocyte-derived leucine aminopeptidase) (A-LAP) (Aminopeptidase PILS) (Puromycin-insensitive leucyl-specific aminopeptidase) (PILS-AP) (Type 1 tumor necrosis factor receptor shedding aminopeptidase regulator)	0.01	9.90E-01	Not Sig.	-0.03	9.84E-01	Not Sig.
O00299	CLIC1	Chloride intracellular channel protein 1 (Chloride channel ABP) (Nuclear chloride ion channel 27) (NCC27) (Regulatory nuclear chloride ion channel protein) (hRNCC)	0.24	5.70E-01	Not Sig.	0.17	6.99E-01	Not Sig.
Q8WUH6	TMEM263	Transmembrane protein 263	0.16	8.20E-01	Not Sig.	0.16	8.17E-01	Not Sig.
P62273	RPS29	40S ribosomal protein S29 (Small ribosomal subunit protein uS14)	-0.21	8.63E-01	Not Sig.	0.67	4.88E-01	Not Sig.
P28838	LAP3	Cytosol aminopeptidase (EC 3.4.11.1) (Cysteinylglycine-S-conjugate dipeptidase) (EC 3.4.13.23) (Leucine aminopeptidase 3) (LAP-3) (Leucyl aminopeptidase) (Peptidase S) (Proline aminopeptidase) (EC 3.4.11.5) (Prolyl aminopeptidase)	-0.27	8.11E-01	Not Sig.	0.75	3.93E-01	Not Sig.
P22087	FBL	rRNA 2'-O-methyltransferase fibrillarin (EC 2.1.1.-) (34 kDa nucleolar scleroderma antigen) (Histone-glutamine methyltransferase) (U6 snRNA 2'-O-methyltransferase fibrillarin)	0.04	9.69E-01	Not Sig.	0.48	4.18E-01	Not Sig.
Q9Y3I0	RTCB	RNA-splicing ligase RtcB homolog (EC 6.5.1.8) (3'-phosphate/5'-hydroxy nucleic acid ligase)	-0.86	4.20E-02	Not Sig.	-0.15	8.14E-01	Not Sig.
P31943	HNRNPH1	Heterogeneous nuclear ribonucleoprotein H (hnRNP H) [Cleaved into: Heterogeneous nuclear ribonucleoprotein H, N-terminally processed]	0.12	7.88E-01	Not Sig.	0.26	4.47E-01	Not Sig.
P02538	KRT6A	Keratin, type II cytoskeletal 6A (Cytokeratin-6A) (CK-6A) (Cytokeratin-6D) (CK-6D) (Keratin-6A) (K6A) (Type-II keratin Kb6) (allergen Hom s 5)	-0.33	8.43E-01	Not Sig.	-1.14	3.49E-01	Not Sig.
P30086	PEBP1	Phosphatidylethanolamine-binding protein 1 (PEBP-1) (HCNPpp) (Neuropolypeptide h3) (Prostatic-binding protein) (Raf kinase inhibitor protein) (RKIP) [Cleaved into: Hippocampal cholinergic neurostimulating peptide (HCNP)]	0.86	7.14E-03	Not Sig.	0.54	1.17E-01	Not Sig.
Q86YZ3	HRNR	Hornerin	0.56	5.03E-01	Not Sig.	-0.18	8.53E-01	Not Sig.
Q6KB66	KRT80	Keratin, type II cytoskeletal 80 (Cytokeratin-80) (CK-80) (Keratin-80) (K80) (Type-II keratin Kb20)	0.61	4.66E-01	Not Sig.	-0.30	7.33E-01	Not Sig.
P49023	PXN	Paxillin	-0.64	3.32E-01	Not Sig.	-0.57	3.92E-01	Not Sig.
Q96IF1	AJUBA	LIM domain-containing protein ajuba	-0.49	4.89E-01	Not Sig.	0.08	9.39E-01	Not Sig.
P22695	UQCRC2	Cytochrome b-c1 complex subunit 2, mitochondrial (Complex III subunit 2) (Core protein II) (Ubiquinol-cytochrome-c reductase complex core protein 2)	0.60	5.88E-02	Not Sig.	-0.10	8.23E-01	Not Sig.
Q15517	CDSN	Corneodesmosin (S protein)	1.10	4.89E-01	Not Sig.	-1.08	4.83E-01	Not Sig.
P46782	RPS5	40S ribosomal protein S5 (Small ribosomal subunit protein uS7) [Cleaved into:	-0.54	6.54E-01	Not Sig.	-0.26	8.23E-01	Not Sig.

		40S ribosomal protein S5, N-terminally processed]						
P43490	NAMPT	Nicotinamide phosphoribosyltransferase (NAmPRTase) (Nampt) (EC 2.4.2.12) (Pre-B-cell colony-enhancing factor 1) (Pre-B cell-enhancing factor) (Visfatin)	-0.39	6.53E-01	Not Sig.	-0.43	5.49E-01	Not Sig.
O43175	PHGDH	D-3-phosphoglycerate dehydrogenase (3-PGDH) (EC 1.1.1.95) (2-oxoglutarate reductase) (EC 1.1.1.399) (Malate dehydrogenase) (EC 1.1.1.37)	-0.50	4.77E-01	Not Sig.	-0.48	4.83E-01	Not Sig.
P31151	S100A7	Protein S100-A7 (Psoriasin) (S100 calcium-binding protein A7)	-0.03	9.90E-01	Not Sig.	0.15	9.48E-01	Not Sig.
P62191	PSMC1	26S proteasome regulatory subunit 4 (P26s4) (26S proteasome AAA-ATPase subunit RPT2) (Proteasome 26S subunit ATPase 1)	-0.57	5.30E-01	Not Sig.	0.22	8.23E-01	Not Sig.
P14625	HSP90B1	Endoplasmin (94 kDa glucose-regulated protein) (GRP-94) (Heat shock protein 90 kDa beta member 1) (Tumor rejection antigen 1) (gp96 homolog)	0.01	9.90E-01	Not Sig.	0.59	2.82E-01	Not Sig.
Q08554	DSC1	Desmocollin-1 (Cadherin family member 1) (Desmosomal glycoprotein 2/3) (DG2/DG3)	1.01	3.89E-01	Not Sig.	-0.30	8.26E-01	Not Sig.
P62244	RPS15A	40S ribosomal protein S15a (Small ribosomal subunit protein uS8)	-0.04	9.52E-01	Not Sig.	0.15	7.13E-01	Not Sig.
P36542	ATP5F1C	ATP synthase subunit gamma, mitochondrial (ATP synthase F1 subunit gamma) (F-ATPase gamma subunit)	-0.26	7.89E-01	Not Sig.	-0.25	7.84E-01	Not Sig.
P30050	RPL12	60S ribosomal protein L12 (Large ribosomal subunit protein uL11)	0.04	9.60E-01	Not Sig.	0.23	6.54E-01	Not Sig.
P02730	SLC4A1	Band 3 anion transport protein (Anion exchange protein 1) (AE 1) (Anion exchanger 1) (Solute carrier family 4 member 1) (CD antigen CD233)	-0.67	6.91E-01	Not Sig.	1.04	4.43E-01	Not Sig.
P31930	UQCRC1	Cytochrome b-c1 complex subunit 1, mitochondrial (Complex III subunit 1) (Core protein I) (Ubiquinol-cytochrome-c reductase complex core protein 1)	0.24	7.59E-01	Not Sig.	-0.02	9.84E-01	Not Sig.
Q9Y305	ACOT9	Acyl-coenzyme A thioesterase 9, mitochondrial (Acyl-CoA thioesterase 9) (EC 3.1.2.-) (Acyl-CoA thioester hydrolase 9)	-0.56	5.56E-01	Not Sig.	-0.04	9.84E-01	Not Sig.
P39019	RPS19	40S ribosomal protein S19 (Small ribosomal subunit protein eS19)	0.59	3.71E-01	Not Sig.	0.32	6.58E-01	Not Sig.
P08708	RPS17	40S ribosomal protein S17 (Small ribosomal subunit protein eS17)	-0.35	7.09E-01	Not Sig.	-0.22	8.17E-01	Not Sig.
P33993	MCM7	DNA replication licensing factor MCM7 (EC 3.6.4.12) (CDC47 homolog) (P1.1-MCM3)	0.17	7.19E-01	Not Sig.	-0.15	7.33E-01	Not Sig.
P29692	EEF1D	Elongation factor 1-delta (EF-1-delta) (Antigen NY-CO-4)	-0.14	9.13E-01	Not Sig.	0.89	1.76E-01	Not Sig.
P60174	TPI1	Triosephosphate isomerase (TIM) (EC 5.3.1.1) (Methylglyoxal synthase) (EC 4.2.3.3) (Triose-phosphate isomerase)	0.21	7.45E-01	Not Sig.	0.73	9.75E-02	Not Sig.
P62913	RPL11	60S ribosomal protein L11 (CLL-associated antigen KW-12) (Large ribosomal subunit protein uL5)	0.04	9.52E-01	Not Sig.	0.15	6.77E-01	Not Sig.
P49959	MRE11	Double-strand break repair protein MRE11 (EC 3.1.-.-) (Double-strand break repair protein MRE11A) (Meiotic recombination 11 homolog 1) (MRE11 homolog 1) (Meiotic recombination 11 homolog A) (MRE11 homolog A)	-0.68	2.83E-01	Not Sig.	0.11	9.06E-01	Not Sig.
Q14669	TRIP12	E3 ubiquitin-protein ligase TRIP12 (EC 2.3.2.26) (E3 ubiquitin-protein ligase for Arf) (ULF) (HECT-type E3 ubiquitin transferase TRIP12) (Thyroid receptor-interacting protein 12) (TR-interacting protein 12) (TRIP-12)	-0.21	8.11E-01	Not Sig.	-0.09	9.32E-01	Not Sig.
P36578	RPL4	60S ribosomal protein L4 (60S ribosomal protein L1) (Large ribosomal subunit protein uL4)	0.09	8.63E-01	Not Sig.	0.20	6.36E-01	Not Sig.
P61513	RPL37A	60S ribosomal protein L37a (Large ribosomal subunit protein eL43)	0.48	6.31E-01	Not Sig.	1.15	9.63E-02	Not Sig.

O15371	EIF3D	Eukaryotic translation initiation factor 3 subunit D (eIF3d) (Eukaryotic translation initiation factor 3 subunit 7) (eIF-3-zeta) (eIF3 p66)	-0.06	9.60E-01	Not Sig.	-0.25	7.59E-01	Not Sig.
P62241	RPS8	40S ribosomal protein S8 (Small ribosomal subunit protein eS8)	0.00	9.94E-01	Not Sig.	0.50	4.01E-01	Not Sig.
P68402	PAFAH1B 2	Platelet-activating factor acetylhydrolase IB subunit alpha2 (EC 3.1.1.47) (PAF acetylhydrolase 30 kDa subunit) (PAF-AH 30 kDa subunit) (PAF-AH subunit beta) (PAFAH subunit beta)	-0.11	9.30E-01	Not Sig.	0.03	9.84E-01	Not Sig.
Q9UQE 7	SMC3	Structural maintenance of chromosomes protein 3 (SMC protein 3) (SMC-3) (Basement membrane-associated chondroitin proteoglycan) (Bamacan) (Chondroitin sulfate proteoglycan 6) (Chromosome-associated polypeptide) (hCAP)	-0.69	3.54E-01	Not Sig.	-0.09	9.37E-01	Not Sig.
P84098	RPL19	60S ribosomal protein L19 (Large ribosomal subunit protein eL19)	0.40	9.49E-02	Not Sig.	0.42	8.89E-02	Not Sig.
Q8IX29	FBXO16	F-box only protein 16	-0.69	6.89E-01	Not Sig.	-0.08	9.83E-01	Not Sig.
P05198	EIF2S1	Eukaryotic translation initiation factor 2 subunit 1 (Eukaryotic translation initiation factor 2 subunit alpha) (eIF-2-alpha) (eIF-2A) (eIF-2alpha)	-0.94	2.72E-01	Not Sig.	0.07	9.71E-01	Not Sig.
Q9Y6W 3	CAPN7	Calpain-7 (EC 3.4.22.-) (PalB homolog) (PalBH)	-0.72	3.78E-01	Not Sig.	0.09	9.48E-01	Not Sig.
P22629		Streptavidin	-0.13	7.45E-01	Not Sig.	-0.08	8.23E-01	Not Sig.
P01040	CSTA	Cystatin-A (Cystatin-AS) (Stefin-A) [Cleaved into: Cystatin-A, N-terminally processed]	-0.03	9.90E-01	Not Sig.	-0.45	7.86E-01	Not Sig.
P33992	MCM5	DNA replication licensing factor MCM5 (EC 3.6.4.12) (CDC46 homolog) (P1-CDC46)	0.18	7.83E-01	Not Sig.	-0.40	4.32E-01	Not Sig.
Q86TB9	PATL1	Protein PAT1 homolog 1 (PAT1-like protein 1) (Protein PAT1 homolog b) (Pat1b) (hPat1b)	0.04	9.60E-01	Not Sig.	0.00	9.96E-01	Not Sig.
O75663	TIPRL	TIP41-like protein (Putative MAPK-activating protein PM10) (Type 2A-interacting protein) (TIP)	-0.80	8.56E-02	Not Sig.	-0.44	4.32E-01	Not Sig.
P59190	RAB15	Ras-related protein Rab-15	-0.65	3.89E-01	Not Sig.	0.14	8.85E-01	Not Sig.
Q9UHB 6	LIMA1	LIM domain and actin-binding protein 1 (Epithelial protein lost in neoplasm)	0.18	8.35E-01	Not Sig.	0.53	4.10E-01	Not Sig.
P62829	RPL23	60S ribosomal protein L23 (60S ribosomal protein L17) (Large ribosomal subunit protein uL14)	0.01	9.90E-01	Not Sig.	-0.17	7.11E-01	Not Sig.
O75131	CPNE3	Copine-3 (Copine III)	0.16	8.11E-01	Not Sig.	0.04	9.71E-01	Not Sig.
P04844	RPN2	Dolichyl-diphosphooligosaccharide--protein glycosyltransferase subunit 2 (Dolichyl-diphosphooligosaccharide--protein glycosyltransferase 63 kDa subunit) (RIBIIR) (Ribophorin II) (RPN-II) (Ribophorin-2)	0.03	9.52E-01	Not Sig.	0.21	4.94E-01	Not Sig.
Q9H6Z4	RANBP3	Ran-binding protein 3 (RanBP3)	0.09	9.52E-01	Not Sig.	0.53	5.37E-01	Not Sig.
P48444	ARCN1	Coatomer subunit delta (Archain) (Delta-coat protein) (Delta-COP)	-0.30	6.92E-01	Not Sig.	0.16	8.23E-01	Not Sig.
P49588	AARS1	Alanine--tRNA ligase, cytoplasmic (EC 6.1.1.7) (Alanyl-tRNA synthetase) (AlaRS) (Renal carcinoma antigen NY-REN-42)	-0.26	7.16E-01	Not Sig.	-0.02	9.84E-01	Not Sig.
P26639	TARS1	Threonine--tRNA ligase 1, cytoplasmic (EC 6.1.1.3) (Threonyl-tRNA synthetase) (ThrRS) (Threonyl-tRNA synthetase 1)	0.17	7.63E-01	Not Sig.	0.34	4.32E-01	Not Sig.
Q9UHD 1	CHORDC 1	Cysteine and histidine-rich domain-containing protein 1 (CHORD domain-containing protein 1) (CHORD-containing protein 1) (CHP-1) (Protein morgana)	-0.19	7.88E-01	Not Sig.	-0.45	4.04E-01	Not Sig.
Q562R1	ACTBL2	Beta-actin-like protein 2 (Kappa-actin)	-0.25	7.09E-01	Not Sig.	0.14	8.23E-01	Not Sig.
Q8IWZ3	ANKHD1	Ankyrin repeat and KH domain-containing protein 1 (HIV-1 Vpr-binding ankyrin repeat protein) (Multiple ankyrin repeats single KH domain) (hMASK)	-0.39	7.05E-01	Not Sig.	0.09	9.48E-01	Not Sig.
P06753	TPM3	Tropomyosin alpha-3 chain (Gamma-tropomyosin) (Tropomyosin-3) (Tropomyosin-5) (hTM5)	-0.41	7.09E-01	Not Sig.	-0.28	7.96E-01	Not Sig.

P16403	H1-2	Histone H1.2 (Histone H1c) (Histone H1d) (Histone H1s-1)	-0.07	9.72E-01	Not Sig.	1.03	2.77E-01	Not Sig.
P46779	RPL28	60S ribosomal protein L28 (Large ribosomal subunit protein eL28)	-0.46	2.53E-01	Not Sig.	-0.95	7.60E-03	Not Sig.
P23490	LORICRIN	Loricrin	0.35	8.63E-01	Not Sig.	-0.72	6.77E-01	Not Sig.
P62888	RPL30	60S ribosomal protein L30 (Large ribosomal subunit protein eL30)	-0.18	7.63E-01	Not Sig.	0.02	9.84E-01	Not Sig.
Q14974	KPNB1	Importin subunit beta-1 (Importin-90) (Karyopherin subunit beta-1) (Nuclear factor p97) (Pore targeting complex 97 kDa subunit) (PTAC97)	0.05	9.73E-01	Not Sig.	0.48	5.49E-01	Not Sig.
Q7Z3Y8	KRT27	Keratin, type I cytoskeletal 27 (Cytokeratin-27) (CK-27) (Keratin-25C) (K25C) (Keratin-27) (K27) (Type I inner root sheath-specific keratin-K25irs3)	0.08	9.59E-01	Not Sig.	0.75	3.93E-01	Not Sig.
P05161	ISG15	Ubiquitin-like protein ISG15 (Interferon-induced 15 kDa protein) (Interferon-induced 17 kDa protein) (IP17) (Ubiquitin cross-reactive protein) (hUCRP)	0.17	9.03E-01	Not Sig.	-0.40	7.11E-01	Not Sig.
O75223	GGCT	Gamma-glutamylcyclotransferase (EC 4.3.2.9) (Cytochrome c-releasing factor 21)	0.87	4.25E-01	Not Sig.	-0.76	4.83E-01	Not Sig.
P29401	TKT	Transketolase (TK) (EC 2.2.1.1)	-0.24	7.88E-01	Not Sig.	0.43	5.37E-01	Not Sig.
Q96B21	TMEM45 B	Transmembrane protein 45B	-0.56	6.89E-01	Not Sig.	0.01	9.96E-01	Not Sig.
Q9NSD 9	FARSB	Phenylalanine--tRNA ligase beta subunit (EC 6.1.1.20) (Phenylalanyl-tRNA synthetase beta subunit) (PheRS)	0.43	6.67E-01	Not Sig.	0.04	9.84E-01	Not Sig.
P31040	SDHA	Succinate dehydrogenase [ubiquinone] flavoprotein subunit, mitochondrial (EC 1.3.5.1) (Flavoprotein subunit of complex II) (Fp)	0.04	9.72E-01	Not Sig.	0.11	8.55E-01	Not Sig.
P61088	UBE2N	Ubiquitin-conjugating enzyme E2 N (EC 2.3.2.23) (Bendless-like ubiquitin-conjugating enzyme) (E2 ubiquitin-conjugating enzyme N) (Ubc13) (UbcH13) (Ubiquitin carrier protein N) (Ubiquitin-protein ligase N)	0.59	5.93E-01	Not Sig.	0.30	7.92E-01	Not Sig.
Q00610	CLTC	Clathrin heavy chain 1 (Clathrin heavy chain on chromosome 17) (CLH-17)	-0.39	4.77E-01	Not Sig.	0.12	8.36E-01	Not Sig.
P60866	RPS20	40S ribosomal protein S20 (Small ribosomal subunit protein uS10)	0.36	3.85E-01	Not Sig.	-0.21	6.43E-01	Not Sig.
P25205	MCM3	DNA replication licensing factor MCM3 (EC 3.6.4.12) (DNA polymerase alpha holoenzyme-associated protein P1) (P1-MCM3) (RLF subunit beta) (p102)	0.52	6.26E-02	Not Sig.	-0.24	4.88E-01	Not Sig.
P21266	GSTM3	Glutathione S-transferase Mu 3 (EC 2.5.1.18) (GST class-mu 3) (GSTM3-3) (hGSTM3-3)	-0.09	9.37E-01	Not Sig.	0.10	9.14E-01	Not Sig.
P14174	MIF	Macrophage migration inhibitory factor (MIF) (EC 5.3.2.1) (Glycosylation-inhibiting factor) (GIF) (L-dopachrome isomerase) (L-dopachrome tautomerase) (EC 5.3.3.12) (Phenylpyruvate tautomerase)	0.20	5.81E-01	Not Sig.	0.41	1.32E-01	Not Sig.
Q5T4S7	UBR4	E3 ubiquitin-protein ligase UBR4 (EC 2.3.2.27) (600 kDa retinoblastoma protein-associated factor) (N-recognin-4) (RING-type E3 ubiquitin transferase UBR4) (Retinoblastoma-associated factor of 600 kDa) (RBAF600) (p600) (Zinc finger UBR1-type protein 1)	0.20	7.63E-01	Not Sig.	0.04	9.71E-01	Not Sig.
Q9NZ01	TECR	Very-long-chain enoyl-CoA reductase (EC 1.3.1.93) (Synaptic glycoprotein SC2) (Trans-2,3-enoyl-CoA reductase) (TER)	0.24	7.88E-01	Not Sig.	0.65	3.36E-01	Not Sig.
P52292	KPNA2	Importin subunit alpha-1 (Karyopherin subunit alpha-2) (RAG cohort protein 1) (SRP1-alpha)	0.50	4.77E-01	Not Sig.	0.43	5.17E-01	Not Sig.
O75533	SF3B1	Splicing factor 3B subunit 1 (Pre-mRNA-splicing factor SF3b 155 kDa subunit) (SF3b155) (Spliceosome-associated protein 155) (SAP 155)	-0.49	6.31E-01	Not Sig.	-1.22	8.89E-02	Not Sig.
O75352	MPDU1	Mannose-P-dolichol utilization defect 1 protein (Suppressor of Lec15 and Lec35 glycosylation mutation homolog) (SL15)	-0.36	6.38E-01	Not Sig.	-0.37	5.75E-01	Not Sig.

Q15323	KRT31	Keratin, type I cuticular Ha1 (Hair keratin, type I Ha1) (Keratin-31) (K31)	-0.08	9.89E-01	Not Sig.	1.30	5.49E-01	Not Sig.
Q16543	CDC37	Hsp90 co-chaperone Cdc37 (Hsp90 chaperone protein kinase-targeting subunit) (p50Cdc37) [Cleaved into: Hsp90 co-chaperone Cdc37, N-terminally processed]	-0.08	8.74E-01	Not Sig.	-0.19	6.29E-01	Not Sig.
Q8N9Q2	SREK1IP1	Protein SREK1IP1 (SFRS12-interacting protein 1) (SREK1-interacting protein 1) (Splicing regulatory protein of 18 kDa) (p18SRP)	-0.29	8.11E-01	Not Sig.	0.60	5.37E-01	Not Sig.
P63092	NA	Guanine nucleotide-binding protein G(s) subunit alpha isoforms short (Adenylate cyclase-stimulating G alpha protein)	0.29	7.77E-01	Not Sig.	-0.31	7.33E-01	Not Sig.
Q13596	SNX1	Sorting nexin-1	0.09	9.30E-01	Not Sig.	-0.50	4.06E-01	Not Sig.
P50454	SERPINH1	Serpin H1 (47 kDa heat shock protein) (Arsenic-transactivated protein 3) (AsTP3) (Cell proliferation-inducing gene 14 protein) (Collagen-binding protein) (Colligin) (Rheumatoid arthritis-related antigen RA-A47)	0.01	9.94E-01	Not Sig.	-0.69	4.94E-01	Not Sig.
Q14697	GANAB	Neutral alpha-glucosidase AB (EC 3.2.1.207) (Alpha-glucosidase 2) (Glucosidase II subunit alpha)	0.13	8.66E-01	Not Sig.	0.37	5.37E-01	Not Sig.
Q8WVV4	POF1B	Protein POF1B (Premature ovarian failure protein 1B)	0.25	8.11E-01	Not Sig.	-0.05	9.83E-01	Not Sig.
Q96AG4	LRRC59	Leucine-rich repeat-containing protein 59 (Ribosome-binding protein p34) (p34) [Cleaved into: Leucine-rich repeat-containing protein 59, N-terminally processed]	-0.01	9.94E-01	Not Sig.	0.17	8.06E-01	Not Sig.
Q15365	PCBP1	Poly(rC)-binding protein 1 (Alpha-CP1) (Heterogeneous nuclear ribonucleoprotein E1) (hnRNP E1) (Nucleic acid-binding protein SUB2.3)	-0.61	3.71E-01	Not Sig.	-0.46	4.94E-01	Not Sig.
P46776	RPL27A	60S ribosomal protein L27a (Large ribosomal subunit protein uL15)	0.05	8.64E-01	Not Sig.	-0.03	9.40E-01	Not Sig.
Q16881	TXNRD1	Thioredoxin reductase 1, cytoplasmic (TR) (EC 1.8.1.9) (Gene associated with retinoic and interferon-induced mortality 12 protein) (GRIM-12) (Gene associated with retinoic and IFN-induced mortality 12 protein) (KM-102-derived reductase-like factor) (Thioredoxin reductase TR1)	-0.38	5.57E-01	Not Sig.	-0.28	6.58E-01	Not Sig.
Q9Y4E8	USP15	Ubiquitin carboxyl-terminal hydrolase 15 (EC 3.4.19.12) (Deubiquitinating enzyme 15) (Ubiquitin thioesterase 15) (Ubiquitin-specific-processing protease 15) (Unph-2) (Unph4)	-1.08	5.01E-02	Not Sig.	-1.12	5.12E-02	Not Sig.
Q13263	TRIM28	Transcription intermediary factor 1-beta (TIF1-beta) (E3 SUMO-protein ligase TRIM28) (EC 2.3.2.27) (KRAB-associated protein 1) (KAP-1) (KRAB-interacting protein 1) (KRIP-1) (Nuclear corepressor KAP-1) (RING finger protein 96) (RING-type E3 ubiquitin transferase TIF1-beta) (Tripartite motif-containing protein 28)	-0.13	8.63E-01	Not Sig.	-0.26	6.77E-01	Not Sig.
O14980	XPO1	Exportin-1 (Exp1) (Chromosome region maintenance 1 protein homolog)	0.19	8.20E-01	Not Sig.	-0.50	4.51E-01	Not Sig.
Q9H910	JPT2	Jupiter microtubule associated homolog 2 (Hematological and neurological expressed 1-like protein) (HN1-like protein)	-0.23	7.64E-01	Not Sig.	-0.70	1.72E-01	Not Sig.
P09493	TPM1	Tropomyosin alpha-1 chain (Alpha-tropomyosin) (Tropomyosin-1)	-0.30	6.89E-01	Not Sig.	0.02	9.84E-01	Not Sig.
P69905	HBA1	Hemoglobin subunit alpha (Alpha-globin) (Hemoglobin alpha chain)	0.29	7.63E-01	Not Sig.	0.35	6.79E-01	Not Sig.
P63220	RPS21	40S ribosomal protein S21 (Small ribosomal subunit protein eS21)	-0.08	9.52E-01	Not Sig.	-0.45	5.17E-01	Not Sig.
Q9UQ80	PA2G4	Proliferation-associated protein 2G4 (Cell cycle protein p38-2G4 homolog) (hG4-1) (ErbB3-binding protein 1)	0.15	7.66E-01	Not Sig.	0.67	5.85E-02	Not Sig.

Q92945	KHSRP	Far upstream element-binding protein 2 (FUSE-binding protein 2) (KH type-splicing regulatory protein) (KSRP) (p75)	0.42	5.57E-01	Not Sig.	0.16	8.32E-01	Not Sig.
Q9ULV4	CORO1C	Coronin-1C (Coronin-3) (hCRNN4)	-0.06	9.60E-01	Not Sig.	-0.21	7.86E-01	Not Sig.
P55036	PSMD4	26S proteasome non-ATPase regulatory subunit 4 (26S proteasome regulatory subunit RPN10) (26S proteasome regulatory subunit S5A) (Antisecretory factor 1) (AF) (ASF) (Multiubiquitin chain-binding protein)	-0.81	7.14E-03	Not Sig.	-0.52	1.14E-01	Not Sig.
P49790	NUP153	Nuclear pore complex protein Nup153 (153 kDa nucleoporin) (Nucleoporin Nup153)	0.32	5.08E-01	Not Sig.	-0.27	5.49E-01	Not Sig.
Q9NZT1	CALML5	Calmodulin-like protein 5 (Calmodulin-like skin protein)	-0.68	4.77E-01	Not Sig.	-0.39	6.99E-01	Not Sig.
Q13428	TCOF1	Treacle protein (Treacher Collins syndrome protein)	0.28	6.86E-01	Not Sig.	0.84	6.95E-02	Not Sig.
Q9Y678	COPG1	Coatomer subunit gamma-1 (Gamma-1-coat protein) (Gamma-1-COP)	-0.17	8.11E-01	Not Sig.	0.19	7.84E-01	Not Sig.
P61313	RPL15	60S ribosomal protein L15 (Large ribosomal subunit protein eL15)	-0.26	6.31E-01	Not Sig.	0.21	6.79E-01	Not Sig.
O14818	PSMA7	Proteasome subunit alpha type-7 (Proteasome subunit RC6-1) (Proteasome subunit XAPC7)	-0.35	7.09E-01	Not Sig.	0.25	7.84E-01	Not Sig.
P42167	NA	Lamina-associated polypeptide 2, isoforms beta/gamma (Thymopoietin, isoforms beta/gamma) (TP beta/gamma) (Thymopoietin-related peptide isoforms beta/gamma) (TPRP isoforms beta/gamma) [Cleaved into: Thymopoietin (TP) (Splenin); Thymopentin (TP5)]	-0.09	9.30E-01	Not Sig.	0.43	5.17E-01	Not Sig.
P42766	RPL35	60S ribosomal protein L35 (Large ribosomal subunit protein uL29)	0.27	5.81E-01	Not Sig.	0.36	4.06E-01	Not Sig.
P07339	CTSD	Cathepsin D (EC 3.4.23.5) [Cleaved into: Cathepsin D light chain; Cathepsin D heavy chain]	0.42	7.84E-01	Not Sig.	-0.66	5.96E-01	Not Sig.
O00571	DDX3X	ATP-dependent RNA helicase DDX3X (EC 3.6.4.13) (CAP-Rf) (DEAD box protein 3, X-chromosomal) (DEAD box, X isoform) (DBX) (Helicase-like protein 2) (HLP2)	-0.34	5.70E-01	Not Sig.	-0.13	8.32E-01	Not Sig.
Q9P035	HACD3	Very-long-chain (3R)-3-hydroxyacyl-CoA dehydratase 3 (EC 4.2.1.134) (3-hydroxyacyl-CoA dehydratase 3) (HACD3) (Butyrate-induced protein 1) (B-ind1) (hB-ind1) (Protein-tyrosine phosphatase-like A domain-containing protein 1)	0.37	7.05E-01	Not Sig.	0.32	7.15E-01	Not Sig.
P09651	HNRNPA 1	Heterogeneous nuclear ribonucleoprotein A1 (hnRNP A1) (Helix-destabilizing protein) (Single-strand RNA-binding protein) (hnRNP core protein A1) [Cleaved into: Heterogeneous nuclear ribonucleoprotein A1, N-terminally processed]	-0.22	7.63E-01	Not Sig.	0.36	5.35E-01	Not Sig.
Q9HCY 8	S100A14	Protein S100-A14 (S100 calcium-binding protein A14) (S114)	-0.48	7.09E-01	Not Sig.	-0.83	4.26E-01	Not Sig.
P26196	DDX6	Probable ATP-dependent RNA helicase DDX6 (EC 3.6.4.13) (ATP-dependent RNA helicase p54) (DEAD box protein 6) (Oncogene RCK)	-0.27	7.64E-01	Not Sig.	0.29	7.31E-01	Not Sig.
O95969	SCGB1D2	Secretoglobin family 1D member 2 (Lipophilin-B)	-0.05	9.86E-01	Not Sig.	-0.27	8.23E-01	Not Sig.
P29218	IMPA1	Inositol monophosphatase 1 (IMP 1) (IMPase 1) (EC 3.1.3.25) (D-galactose 1-phosphate phosphatase) (EC 3.1.3.94) (Inositol-1(or 4)-monophosphatase 1) (Lithium-sensitive myo-inositol monophosphatase A1)	-0.14	9.14E-01	Not Sig.	-0.11	9.27E-01	Not Sig.
Q96CW 1	AP2M1	AP-2 complex subunit mu (AP-2 mu chain) (Adaptin-mu2) (Adaptor protein complex AP-2 subunit mu) (Adaptor-related protein complex 2 subunit mu) (Clathrin assembly protein	-0.41	5.47E-01	Not Sig.	0.02	9.84E-01	Not Sig.

		complex 2 mu medium chain) (Clathrin coat assembly protein AP50) (Clathrin coat-associated protein AP50) (HA2 50 kDa subunit) (Plasma membrane adaptor AP-2 50 kDa protein)						
Q8TDY2	RB1CC1	RB1-inducible coiled-coil protein 1 (FAK family kinase-interacting protein of 200 kDa) (FIP200)	-0.59	4.54E-01	Not Sig.	0.00	9.99E-01	Not Sig.
Q9UNF1	MAGED2	Melanoma-associated antigen D2 (11B6) (Breast cancer-associated gene 1 protein) (BCG-1) (Hepatocellular carcinoma-associated protein JCL-1) (MAGE-D2 antigen)	-0.14	9.09E-01	Not Sig.	-0.52	5.06E-01	Not Sig.
Q8TF72	SHROOM3	Protein Shroom3 (Shroom-related protein) (hShrmL)	-0.43	7.63E-01	Not Sig.	-0.20	8.93E-01	Not Sig.
P61626	LYZ	Lysozyme C (EC 3.2.1.17) (1,4-beta-N-acetylmuramidase C)	-0.49	8.11E-01	Not Sig.	-0.61	7.49E-01	Not Sig.
Q8N823	ZNF611	Zinc finger protein 611	-0.48	6.91E-01	Not Sig.	-0.04	9.84E-01	Not Sig.
P63104	YWHAZ	14-3-3 protein zeta/delta (Protein kinase C inhibitor protein 1) (KCIP-1)	-0.31	8.06E-01	Not Sig.	0.76	4.31E-01	Not Sig.
P48047	ATP5PO	ATP synthase subunit O, mitochondrial (ATP synthase peripheral stalk subunit OSCP) (Oligomycin sensitivity conferral protein) (OSCP)	-0.20	8.28E-01	Not Sig.	0.35	6.63E-01	Not Sig.
P15170	GSPT1	Eukaryotic peptide chain release factor GTP-binding subunit ERF3A (Eukaryotic peptide chain release factor subunit 3a) (eRF3a) (G1 to S phase transition protein 1 homolog)	-0.25	7.77E-01	Not Sig.	-0.34	6.63E-01	Not Sig.
O60664	PLIN3	Perilipin-3 (47 kDa mannose 6-phosphate receptor-binding protein) (47 kDa MPR-binding protein) (Cargo selection protein TIP47) (Mannose-6-phosphate receptor-binding protein 1) (Placental protein 17) (PP17)	-0.42	4.57E-01	Not Sig.	-0.69	1.15E-01	Not Sig.
A6NHQ2	FBLL1	rRNA/tRNA 2'-O-methyltransferase fibrillarin-like protein 1 (EC 2.1.1.-) (Protein-glutamine methyltransferase)	-0.60	3.71E-01	Not Sig.	0.14	8.57E-01	Not Sig.
O94979	SEC31A	Protein transport protein Sec31A (ABP125) (ABP130) (SEC31-like protein 1) (SEC31-related protein A) (Web1-like protein)	-0.33	7.63E-01	Not Sig.	0.35	7.31E-01	Not Sig.
Q9H3U1	UNC45A	Protein unc-45 homolog A (Unc-45A) (GCUNC-45) (Smooth muscle cell-associated protein 1) (SMAP-1)	-0.68	2.97E-01	Not Sig.	-0.56	3.95E-01	Not Sig.
Q6ZVX7	NCCRP1	F-box only protein 50 (NCC receptor protein 1 homolog) (NCCRP-1) (Non-specific cytotoxic cell receptor protein 1 homolog)	0.84	3.15E-01	Not Sig.	-0.66	4.35E-01	Not Sig.
O43684	BUB3	Mitotic checkpoint protein BUB3	0.28	6.83E-01	Not Sig.	0.01	9.86E-01	Not Sig.
Q13838	DDX39B	Spliceosome RNA helicase DDX39B (EC 3.6.4.13) (56 kDa U2AF65-associated protein) (ATP-dependent RNA helicase p47) (DEAD box protein UAP56) (HLA-B-associated transcript 1 protein)	-0.11	8.84E-01	Not Sig.	0.05	9.66E-01	Not Sig.
Q05639	EEF1A2	Elongation factor 1-alpha 2 (EF-1-alpha-2) (Eukaryotic elongation factor 1 A-2) (eEF1A-2) (Statin-S1)	-0.01	9.89E-01	Not Sig.	0.09	8.55E-01	Not Sig.
O14979	HNRNPDL	Heterogeneous nuclear ribonucleoprotein D-like (hnRNP D-like) (hnRNP DL) (AU-rich element RNA-binding factor) (JKT41-binding protein) (Protein laAUF1)	0.39	5.30E-01	Not Sig.	-0.39	4.94E-01	Not Sig.
P46977	STT3A	Dolichyl-diphosphooligosaccharide--protein glycosyltransferase subunit STT3A (Oligosaccharyl transferase subunit STT3A) (STT3-A) (EC 2.4.99.18) (B5) (Integral membrane protein 1) (Transmembrane protein TMC)	-0.44	4.77E-01	Not Sig.	-0.17	8.14E-01	Not Sig.
O60884	DNAJA2	DnaJ homolog subfamily A member 2 (Cell cycle progression restoration gene 3 protein) (Dnj3) (Dj3) (HIRA-interacting protein 4) (Renal carcinoma antigen NY-REN-14)	-0.63	4.54E-01	Not Sig.	-1.09	9.23E-02	Not Sig.

Q9Y496	KIF3A	Kinesin-like protein KIF3A (Microtubule plus end-directed kinesin motor 3A)	-0.51	6.65E-01	Not Sig.	-0.15	9.10E-01	Not Sig.
P49591	SARS1	Serine--tRNA ligase, cytoplasmic (EC 6.1.1.11) (Seryl-tRNA synthetase) (SerRS) (Seryl-tRNA(Ser/Sec) synthetase)	-0.41	5.70E-01	Not Sig.	0.60	3.06E-01	Not Sig.
P19623	SRM	Spermidine synthase (SPDSY) (EC 2.5.1.16) (Putrescine aminopropyltransferase)	0.70	1.69E-01	Not Sig.	0.00	9.96E-01	Not Sig.
P05090	APOD	Apolipoprotein D (Apo-D) (ApoD)	-0.41	7.34E-01	Not Sig.	-0.10	9.53E-01	Not Sig.
P13489	RNH1	Ribonuclease inhibitor (Placental ribonuclease inhibitor) (Placental RNase inhibitor) (Ribonuclease/angiogenin inhibitor 1) (RAI)	-0.19	8.11E-01	Not Sig.	0.41	5.37E-01	Not Sig.
Q99497	PARK7	Parkinson disease protein 7 (Maillard deglycase) (Oncogene DJ1) (Parkinsonism-associated deglycase) (Protein DJ-1) (DJ-1) (Protein/nucleic acid deglycase DJ-1) (EC 3.1.2.-) (EC 3.5.1.-) (EC 3.5.1.124)	-0.25	7.09E-01	Not Sig.	0.01	9.84E-01	Not Sig.
E9PRG8	C11orf98	Uncharacterized protein C11orf98	-0.58	4.93E-01	Not Sig.	1.14	8.89E-02	Not Sig.
Q9UHD8	SEPTIN9	Septin-9 (MLL septin-like fusion protein MSF-A) (MLL septin-like fusion protein) (Ovarian/Breast septin) (Ov/Br septin) (Septin D1)	-0.02	9.90E-01	Not Sig.	-0.30	7.34E-01	Not Sig.
P53618	COPB1	Coatomer subunit beta (Beta-coat protein) (Beta-COP)	-0.50	4.77E-01	Not Sig.	0.42	5.35E-01	Not Sig.
P63173	RPL38	60S ribosomal protein L38 (Large ribosomal subunit protein eL38)	0.04	9.72E-01	Not Sig.	0.59	1.95E-01	Not Sig.
P78347	GTF2I	General transcription factor II-I (GTFII-I) (TFII-I) (Bruton tyrosine kinase-associated protein 135) (BAP-135) (BTK-associated protein 135) (SRF-Phox1-interacting protein) (SPIN) (Williams-Beuren syndrome chromosomal region 6 protein)	0.63	4.38E-03	Not Sig.	-0.42	8.25E-02	Not Sig.
