# Supplementary material for: Caspase-2 protects against ferroptotic cell death
Source: Cell Death Dis. 2024 Mar 1;15(3):182. doi: 10.1038/s41419-024-06560-6 (PMC10907636; doi:10.1038/s41419-024-06560-6)
Supplement: Supplementary file 8 — Supplementary Table 2 [file 41419_2024_6560_MOESM8_ESM.pdf]

Supplementary table 2

The acquisition scheme for diaPASEF

MS Type	Cycle Id	Start IM [1/K0]	End IM [1/K0]	Start Mass [m/z]	End Mass [m/z]	CE [eV]
MS1	0	-	-	-	-	-
PASEF	1	0.9001	1.2001	800.00	826.00	-
PASEF	1	0.6000	0.9001	400.00	426.00	-
PASEF	2	0.9201	1.2201	825.00	851.00	-
PASEF	2	0.6200	0.9201	425.00	451.00	-
PASEF	3	0.9301	1.2301	850.00	876.00	-
PASEF	3	0.6300	0.9301	450.00	476.00	-
PASEF	4	0.9500	1.2501	875.00	901.00	-
PASEF	4	0.6501	0.9500	475.00	501.00	-
PASEF	5	0.9600	1.2601	900.00	926.00	-
PASEF	5	0.6601	0.9600	500.00	526.00	-
PASEF	6	0.9800	1.2801	925.00	951.00	-
PASEF	6	0.6801	0.9800	525.00	551.00	-
PASEF	7	0.9900	1.2901	950.00	976.00	-
PASEF	7	0.6900	0.9900	550.00	576.00	-
PASEF	8	1.0101	1.3101	975.00	1001.00	-
PASEF	8	0.7100	1.0101	575.00	601.00	-
PASEF	9	1.0201	1.3201	1000.00	1026.01	-
PASEF	9	0.7200	1.0201	600.00	626.00	-
PASEF	10	1.0401	1.3401	1025.01	1051.01	-
PASEF	10	0.7400	1.0401	625.00	651.00	-
PASEF	11	1.0601	1.3601	1050.01	1076.01	-
PASEF	11	0.7601	1.0601	650.00	676.00	-
PASEF	12	1.0701	1.3701	1075.01	1101.01	-
PASEF	12	0.7701	1.0701	675.00	701.00	-
PASEF	13	1.0901	1.3901	1100.01	1126.01	-
PASEF	13	0.7901	1.0901	700.00	726.00	-
PASEF	14	1.1001	1.4001	1125.01	1151.01	-
PASEF	14	0.8001	1.1001	725.00	751.00	-
PASEF	15	1.1201	1.4201	1150.01	1176.01	-
PASEF	15	0.8200	1.1201	750.00	776.00	-
PASEF	16	1.1300	1.4301	1175.01	1201.01	-
PASEF	16	0.8300	1.1300	775.00	801.00	-
