# Supplementary material for: Caspase-2 protects against ferroptotic cell death
Source: Cell Death Dis. 2024 Mar 1;15(3):182. doi: 10.1038/s41419-024-06560-6 (PMC10907636; doi:10.1038/s41419-024-06560-6)

Original western blots

Figure 1a

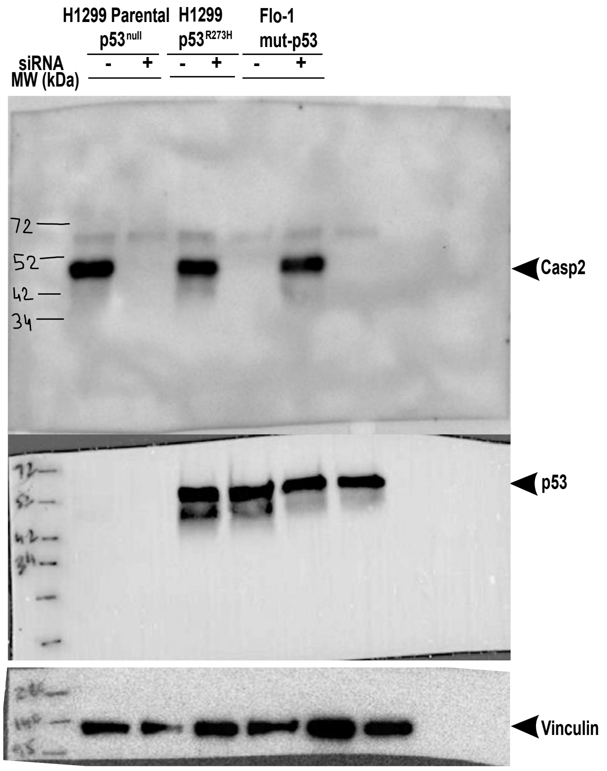

Figure 2a

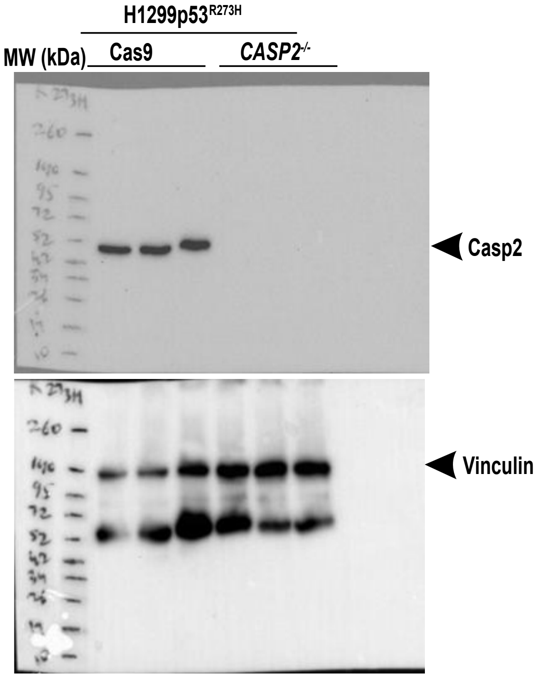

Figure 4

b)

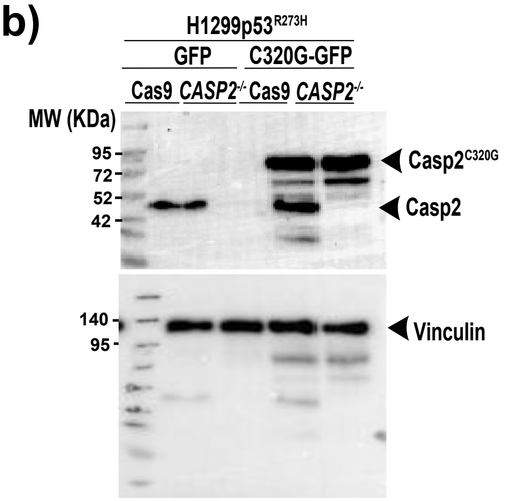

H1299p53R273H
GFP
C320G-GFP
Cas9
CASP2-/-
Cas9
CASP2-/-
MW (KDa)
95
72
52
42
140
95
Casp2C320G
Casp2
Vinculin

d)

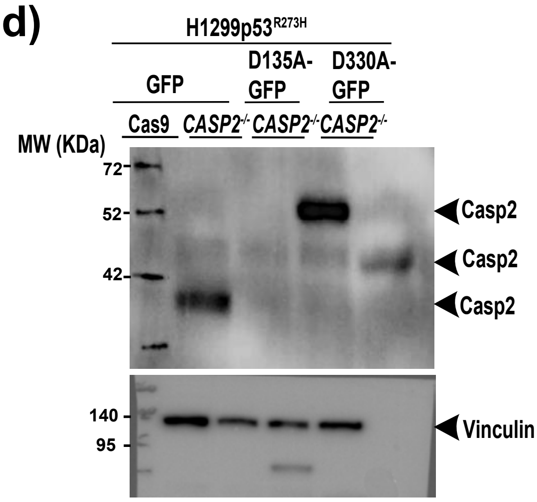

H1299p53R273H
GFP
D135A-GFP
D330A-GFP
Cas9
CASP2-/-
CASP2-/-
CASP2-/-
MW (KDa)
72
52
42
140
95
Casp2
Casp2
Casp2
Vinculin

Figure 6

a)

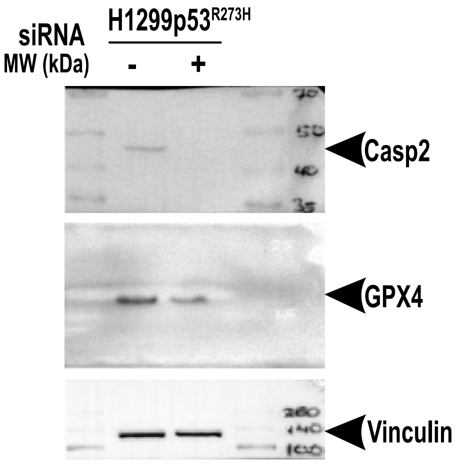

siRNA
MW (kDa)
H1299p53R273H
-
+
Casp2
GPX4
Vinculin

c)

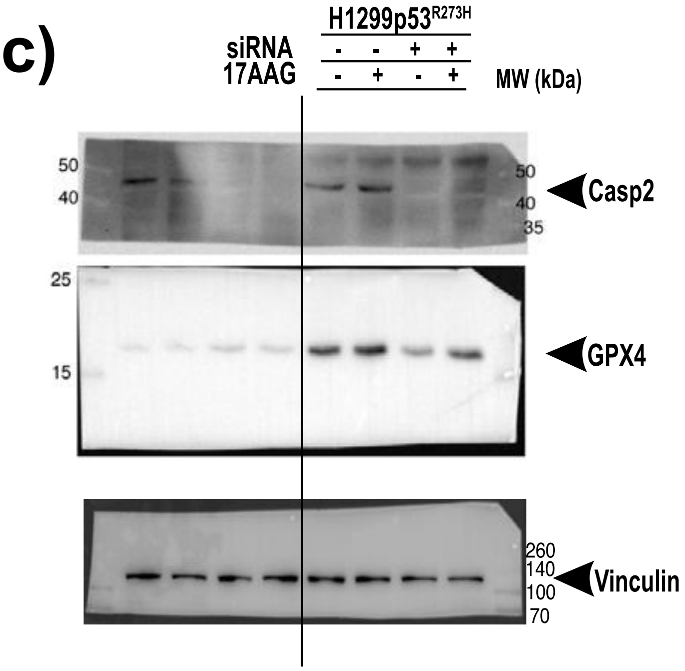

H1299p53R273H
siRNA
17AAG
- - + +
- + - +
MW (kDa)
50
40
50
40
35
Casp2
25
15
GPX4
260
140
100
70
Vinculin

Supplementary Figure S1

g)

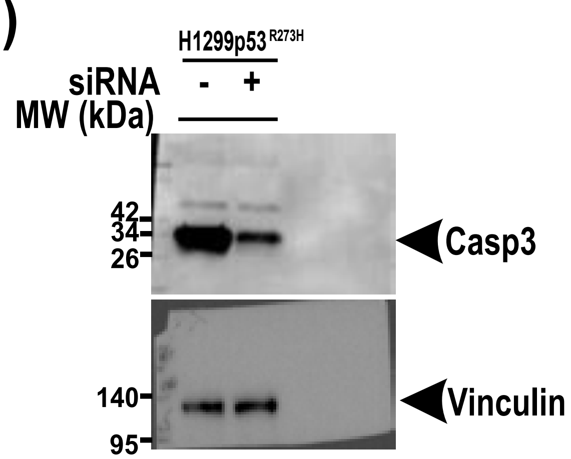

Supplementary Figure S2

a)

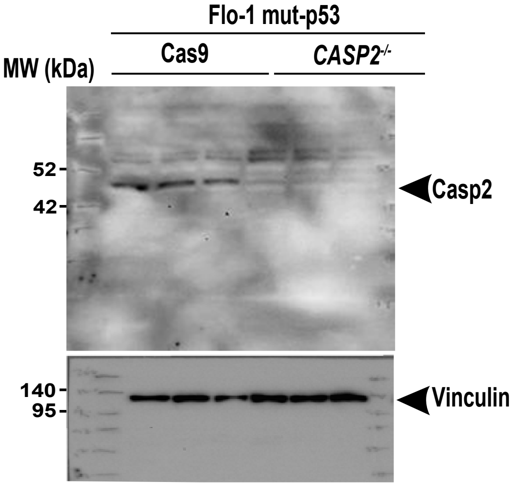

g)

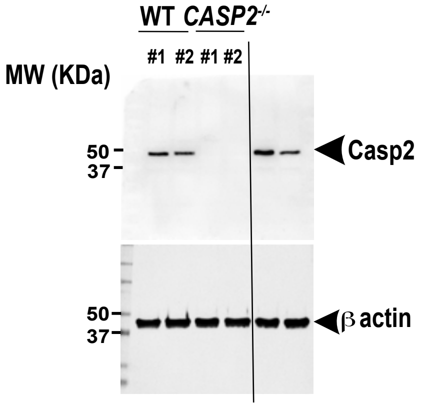

Supplementary Figure S4

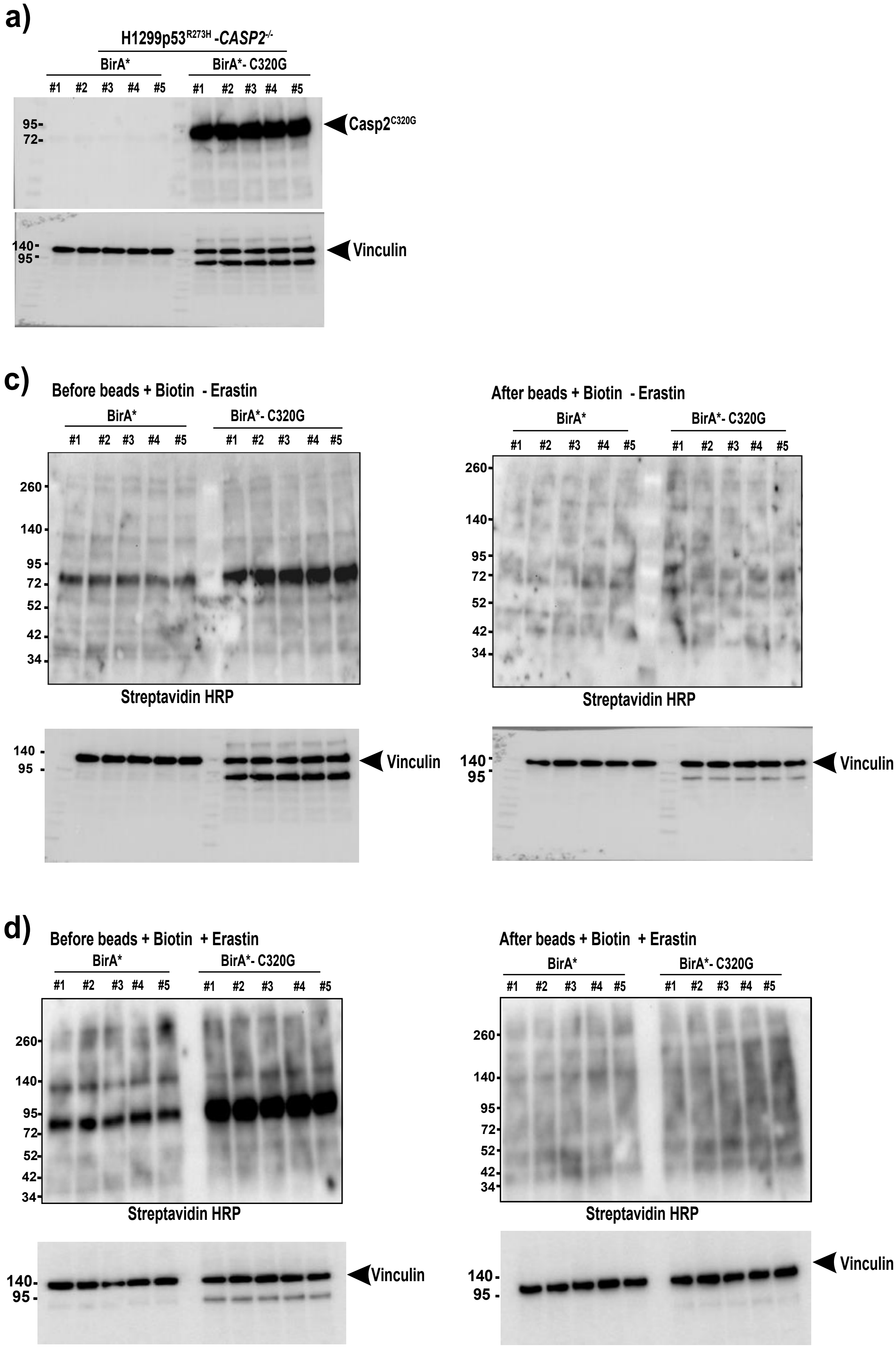

Supplementary Figure S5

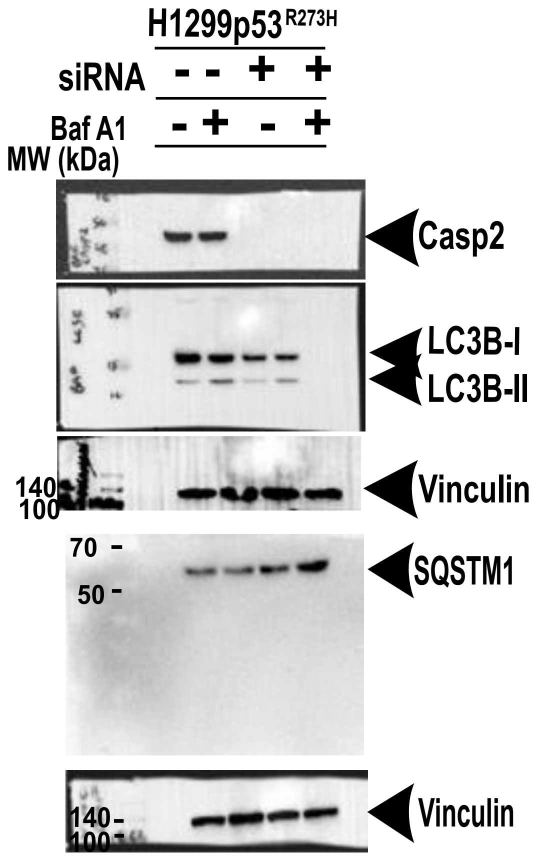

Supplement: Supplementary file 13 — Original western blots [file 41419_2024_6560_MOESM13_ESM.pdf]
